# Comprehensive transcriptome analysis reveals altered mRNA splicing and post-transcriptional changes in the aged mouse brain

**DOI:** 10.1093/nar/gkae172

**Published:** 2024-03-12

**Authors:** Nisha Hemandhar Kumar, Verena Kluever, Emanuel Barth, Sebastian Krautwurst, Mattia Furlan, Mattia Pelizzola, Manja Marz, Eugenio F Fornasiero

**Affiliations:** Department of Neuro- and Sensory Physiology, University Medical Center Göttingen, 37073 Göttingen, Germany; Department of Neuro- and Sensory Physiology, University Medical Center Göttingen, 37073 Göttingen, Germany; Faculty of Mathematics and Computer Science, Friedrich Schiller University Jena, 07743 Jena, Germany; Bioinformatics Core Facility, Friedrich Schiller University Jena, 07743 Jena, Germany; Faculty of Mathematics and Computer Science, Friedrich Schiller University Jena, 07743 Jena, Germany; Center for Genomic Science of IIT@SEMM, Fondazione Istituto Italiano di Tecnologia (IIT), 20139 Milan, Italy; Center for Genomic Science of IIT@SEMM, Fondazione Istituto Italiano di Tecnologia (IIT), 20139 Milan, Italy; Department of Biotechnology and Biosciences, University of Milano-Bicocca, 20126 Milan, Italy; Faculty of Mathematics and Computer Science, Friedrich Schiller University Jena, 07743 Jena, Germany; Leibniz Institute for Age Research, FLI, Beutenbergstraße 11, Jena 07743, Germany; European Virus Bioinformatics Center, Friedrich Schiller University, Leutragraben 1, Jena 07743, Germany; German Center for Integrative Biodiversity Research (iDiv), Puschstraße 4, Leipzig 04103, Germany; Michael Stifel Center Jena, Friedrich Schiller University, Ernst-Abbe-Platz 2, Jena 07743, Germany; Cluster of Excellence Balance of the Microverse, Friedrich Schiller University, Fuerstengraben 1, Jena 07743, Germany; Department of Neuro- and Sensory Physiology, University Medical Center Göttingen, 37073 Göttingen, Germany; Department of Life Sciences, University of Trieste, 34127 Trieste, Italy

## Abstract

A comprehensive understanding of molecular changes during brain aging is essential to mitigate cognitive decline and delay neurodegenerative diseases. The interpretation of mRNA alterations during brain aging is influenced by the health and age of the animal cohorts studied. Here, we carefully consider these factors and provide an in-depth investigation of mRNA splicing and dynamics in the aging mouse brain, combining short- and long-read sequencing technologies with extensive bioinformatic analyses. Our findings encompass a spectrum of age-related changes, including differences in isoform usage, decreased mRNA dynamics and a module showing increased expression of neuronal genes. Notably, our results indicate a reduced abundance of mRNA isoforms leading to nonsense-mediated RNA decay and suggest a regulatory role for RNA-binding proteins, indicating that their regulation may be altered leading to the reshaping of the aged brain transcriptome. Collectively, our study highlights the importance of studying mRNA splicing events during brain aging.

## Graphical Abstract

**Graphical Abstract:**
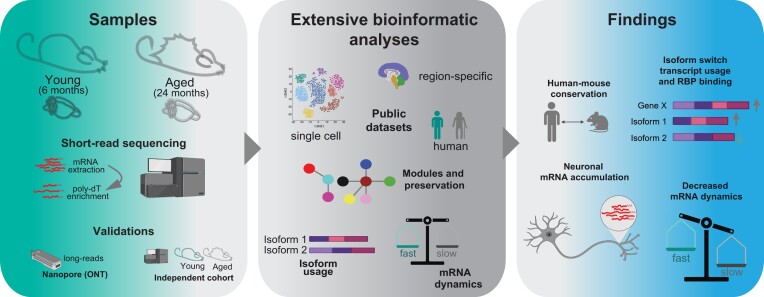

## Introduction

Brain aging is a universal phenomenon associated with cognitive decline and progressive deterioration of physiological and biochemical processes (1). With advancing age, the functional capacity of the human brain gradually deteriorates, resulting in loss of attention, reduced efficiency of sensory perception, and lack of coordination (2). Age-related cognitive impairment is associated with cellular and anatomical changes, such as decreased myelination (3) and synaptic alterations (4). Importantly, age is the major risk factor for neurodegenerative pathologies such as Alzheimer's and Parkinson's disease (5), making the study of the molecular mechanisms underlying aging crucial to preventing disease and maximizing human healthspan.

In the past, a number of gene expression profiling studies have been performed in the brain, comparing the transcriptomes of younger and older brains very often using mice as model organism (6–19). The main finding of these studies is that with aging there is an upregulation of genes involved in complement activation and immune function (20–23), evidenced by upregulation of microglial and astrocyte genes (21) and downregulation of genes encoding immunosuppressive factors⁠ (24). In addition, astrocytes upregulate the expression of inflammatory factors that can lead to synaptic damage (24)⁠. The increase in the immune response has also been observed in human brain aging (4). although in patients it is challenging to distinguish physiological brain aging from cases where the over-activated immune environment actually corresponds to a very early step in the progression of neurodegenerative changes (25).

At the molecular level, brain aging comprises several alterations such as changes in DNA accessibility and histone post-translational modification patterns⁠, resulting in long-term alterations in expression profiles of neurons and glial cells (26). Recent data have also highlighted the importance of DNA repair in neurons (27) and the role in transcriptional elongation fidelity (28) suggesting that the mechanisms upholding the integrity of the transcriptome and proteome face heightened vulnerability during the aging process. Remarkably, the changes observed in protein levels within the aging brain are subtle (29) ^⁠^with the predominant alterations revolving around shifts in protein aggregation (30)^⁠^ and protein turnover profiles (31). The seemingly low extent of proteomic changes might suggest that overall molecular changes in the brain are limited. It could also imply that protective buffering mechanisms counteract transcriptomic changes at the proteomic level. However, the reality is more complex.

Conventional liquid chromatography-mass spectrometry-based techniques employed in proteomics depend on bottom-up strategies, deconstructing proteins into peptides for subsequent in-silico protein identification via matching with a protein database (32). This methodology inherently forfeits information about protein isoforms, a challenge further complicated by the presence of posttranslational modifications and diverse proteoforms (33). While top-down proteomic approaches capable of surmounting these limitations exist and are progressively becoming more accessible (34–36)⁠, their implementation is not without complexity, and their coverage of the proteome remains moderate. In the pursuit of investigating cellular diversity, recent years have witnessed the emergence of various single-cell transcriptomic methodologies (37–39) enabling the comprehensive profiling of entire cell populations within the aged brain (6). These foundational works have supplied a wealth of information, shedding light on the activation of glial and immune cells during aging. They significantly contribute to our understanding of the aging process in the brain. However, the rapid expansion of these fields has not yet rendered them applicable to an exhaustive analysis of isoform discrepancies, due to the limited number of genes and proteins that can be captured by these methodologies (40,41).

Changes in isoforms at the mRNA level result from alternative splicing (AS), a process that generates multiple molecules from a single gene, resulting in a vast functional diversity and tissue complexity that makes the study of transcriptomes fascinating in the post-genomic era (42–45). In addition to potentially encoding different proteins, different mRNA isoforms can affect mRNA stability by altering the interaction of RNA-binding proteins (RBPs). Moreover, adjustments in the length of the 3′-untranslated region (3′UTR) can regulate translation rate and thus modulate protein levels (46–48). Furthermore, differences in isoform expression diversity could be based on variable usage of untranslated transcripts and/or non-principal isoforms, implying that even small changes in isoform usage could significantly affect downstream proteome function (49).

Strikingly, the brain has the highest level of age-related splicing changes compared to most of the other organs in the body (43)^⁠^. Age-related diseases such as Parkinson's disease (7), Alzheimer's disease (50), and frontotemporal lobar dementia (51) are associated with changes in RNA metabolism, particularly mRNA splicing. Amyotrophic lateral sclerosis (52) and autism (53) have been linked to mutations in RNA-binding proteins involved in splicing control and aberrant splicing. Splicing factors such as the polypyrimidine tract binding proteins 1 and 2 (PTBP1 and PTBP2) and other RBPs such as the heterogeneous nuclear ribonuclear proteins A1, H1, H3, and F (hnRNPA1, hnRNPH1, hnRNPH3, hnRNPF) show increased mRNA levels with age in humans (54), suggesting that a tight crosstalk between mRNA species and RBPs is necessary to regulate these pathways. These observations suggest that transcription factors and RBPs may potentially affect exon/intron inclusion (4,55) and may play an important role in age-dependent changes.

In light of these considerations, although isoform changes have been studied in the context of brain aging (54,56,57), none of the previous work has extensively analyzed differential transcript usage (DTU) during brain aging, and systematically addressed the general aspects that control this process. This is crucial given the current insufficient exploration of this field (57–61), and has the potential to pave the way for novel discoveries also in the light of the recent development of technologies that allow to systematically study the role of RBPs (62,63). Thus, here we have chosen to focus our efforts on the comprehensive analysis of the overarching mechanisms that govern isoform transitions and DTU in the context of brain aging. We concentrated on whole brain bulk transcriptome analysis, and used two sequencing technologies, short-read RNAseq and long-read RNAseq acquired through Oxford Nanopore technology (ONT). We utilized brain samples from both young adult (6 months) and aged adult (24 months) male mice and analyzed two independent cohorts of mice to obtain reproducible and accurate data. Our approach encompassed the examination of mRNA expression, extensive clustering, scrutiny of isoform usage, RBP binding probability and a study of RNA dynamics. We have also extensively integrated our results with re-analyses of recent works using complementary approaches such as single-cell sequencing. Our work contributes to a clearer understanding of changes due to aging, identifies a specific pattern in the expression of genes showing a switch between isoforms bidirectionally expressed and highlights a difference in mRNA dynamics during brain aging. This multifaceted analysis offered a comprehensive view of the transcriptomic landscape in the aging brain.

## Materials and methods

### Methodological overview

We used bulk RNA sequencing from two cohorts of male mice at 6 and 24 months of age to explore the changes in mRNA expression, isoform usage, and post-transcriptional RNA dynamics during brain aging. RNA was extracted from the whole brain and subjected to poly-A enrichment.

We concentrated on males as we have done in the past (31,64) as it has been proposed that gene expression changes associated with brain aging are more apparent in males than females (65) and studying male mice allowed us to evaluate the specific effect of genes expressed on the Y-chromosome. We generated short-read RNAseq data and in parallel acquired long-read transcriptome data using Oxford nanopore sequencing technology (ONT) to strengthen our results and cross-validate them with a different sequencing technology. We also added a second cohort of animals for biological validation purposes. Unless otherwise stated, downstream analyses presented here were primarily based on gene or isoform quantification from the original short-read paired-end data dataset. We generated ∼130M reads per sample for the short-read paired-end RNAseq dataset (average read length of 151 bp), ∼30M reads per sample for the short-read single-end RNAseq dataset (average read length of 50 bp) and ∼1M reads per sample for the long-read dataset (average read length of 825 bp; Supplementary Table S1). As expected by the long-read dataset, the read length ranges from ∼140–50 000 bp, which is common for nanopore datasets (66). In both cases, raw reads were processed and mapped to the reference genome (GRm39.103) and were quantified at the gene and at the isoform level. Further downstream analyses were performed to explore differences across ages.

### Animal cohorts

All performed animal experiments were approved by the local authority, the Lower Saxony State Office for Consumer Protection and Food Safety (Niedersächsisches Landesamt für Verbraucherschutz und Lebensmittelsicherheit). The cohorts of mice used here (C57Bl/6JRj) were purchased from Janvier labs and routinely checked for absence of the following four classes of pathogens: (i) bacteria and fungi: *Bordetella bronchiseptica*, CAR bacillus, *Citrobacter rodentium*, *Clostridium piliforme*, *Corynebacterium bovis*, *Corynebacterium kutscheri*, *Dermatophytes*, *Encephalitozoon cuniculi*, *Helicobacter* spp, *Mycoplasma pulmonis*, *Pasteurellaceae*, *Actinobacillus* spp., *Haemophilus* spp., *Mannheimia haemolytica*, *Pasteurella* spp., *Pasteurella multocida*, *Pasteurella pneumotropica*, *Pasteurella trehalosi*, *Salmonella* spp., *Streptobacillus moniliformis*, *Streptococci ß-hemolytic* (not group D), *Streptococcus pneumoniae*; (ii) endoparasites: protozoa, *Entamoeba* spp., Flagellates, Coccidia, Helminths, Cestodes, Nematodes; (iii) ectoparasites: mites, fur-dwelling mites, surface-dwelling mites, follicle-dwelling mites, lice, fleas; (iv) viruses: hantaviruses, K virus (mouse pneumonitis virus), lactate deshydrogenase elevating virus (LDV), lymphocitic choriomenigitis virus (LCMV), minute virus of mice (MVM), mouse adenovirus (MAD) type 1 (FL), mouse adenovirus (MAD) type 2 (K87), mouse cytomegalovirus (MCMV), mouse hepatitis virus (MHV), mouse parvovirus (MPV), mouse polyomavirus, mouse rotavirus (EDIM), mouse thymic virus (MTV), mousepox (ectromelia) virus, murine norovirus (MNV), pneumonia virus of mice, reovirus type 3 (Reo 3), Sendai virus, Theiler's murine encephalomyelitis virus (TMEV).

### Tissue isolation and mRNA extraction

For mRNA isolation, whole hemispheres were dissected from two cohorts of four male mice (four animal replicates for two cohorts, eight adult and eight aged in total) from each age group (young-adult 6m and aged-adult 24m) and further used for RNAseq analysis. After rapid dissection on ice, the tissue was washed briefly in 320 mM sucrose buffer (320 mM sucrose, 5 mM HEPES pH 7.4) and homogenized with a Teflon pestle in glass homogenizer operated with a drill (10 strokes at 900 rpm) in 2 ml of sucrose buffer supplemented with RNase inhibitors (New England Biolabs, cat. M0314, at the concentrations indicated by the manufacturer). This step allowed to obtain a homogenous tissue lysate representative for the whole brain. The lysate (500 μl) was used for RNA extraction, mixed with 800 μl Trizol and extracted with the Qiagen RNeasy mini kit following the manufacturer's protocol. Total brain mRNA was always kept on ice and stored at –80°C.

### Library preparation and sequencing for short-read dataset

Sequencing of RNA samples from a first biological cohort of 8 male mice was performed using Illumina's next-generation sequencing methodology (67). In detail, total RNA was quantified, and quality checked using the Tapestation 4200 instrument in combination with RNA ScreenTape (both Agilent Technologies). Libraries were prepared from 500 ng of input material (total RNA) using NEBNext Ultra II Directional RNA Library Preparation Kit in combination with NEBNext Poly(A) mRNA Magnetic Isolation Module and NEBNext Multiplex Oligos for Illumina (96 Unique Dual Index Primer Pairs) following the manufacturer's instructions (New England Biolabs). Quantification and quality checks of libraries was done using an Agilent 4200 Tapestation Instrument and D1000 ScreenTapes (Agilent Technologies). Libraries were pooled and sequenced on a NovaSeq 6000 SP 300 cycle run. System runs in 151 cycle/paired-end/standard loading workflow mode. Sequence information was converted to FASTQ format using bcl2fastq v2.20.0.422. The datasets generated and analyzed for the short-read dataset are available on GEO under the accession number: GSE233835. For validation purposes, we analyzed samples from a second independent cohort of 8 male mice. This second short-read dataset is available in GEO under accession number GSE249499 as a subseries of our main series GSE233837 (see data availability section). In this case, the samples were processed in a different sequencing facility according to the protocol for Illumina stranded mRNA preparation and the mRNAs were sequenced on the HiSeq4000 with a length of 50 bp (single-end) in order to exclude a possible bias due to where the samples were processed and sequenced. Sequencing was performed by the Transcriptome and Genome Analysis Laboratory (Göttingen).

### Short-read data quality controls and alignment

First, the ‘fastq’ files were evaluated for read quality using the FastQC v0.11.9 (68) through the command line tool. The adapter sequence (‘AGATCGGAAGAGC’) from the ‘fastq’ files was trimmed using trimgalore v0.4.11 (69) and these trimmed fastq files were assessed for read quality using the FastQC v0.11.9 command line tool (68). Second, the ‘fastq’ files were mapped to the reference mouse genome (GRCm39.103) using the ‘hisat2’ tool (70) and quantified using feature counts from subread package (71) for gene-level read counts. Note that for isoform-level read counts, the raw files were mapped and quantified to the transcriptome using salmon v0.10.2 (72). Third, gene and isoform reads were filtered for low counts (≤5 reads) and quantile normalization followed by row-wise median normalization. This allowed us to compare these datasets more reliably across the biological replicates. The number of splicing isoforms was determined in either the young adult or aged brain after applying a minimum cutoff (>2) to check if there was a difference in the number of splicing isoforms between the ages. The normalized data files were visualized with Principal Component Analysis (PCA) in R using prcomp() function to identify possible outliers. As these were not found, we proceeded with further analyses.

### Oxford nanopore technology (ONT) sequencing

Sample measurements using RNA High Sensitivity (HS) assay kit were taken before the library preparation for Direct RNA nanopore sequencing was started, and cleanup and concentration of the sample were performed. The samples from the first cohort of male mice (matched to the subseries GSE233835) were measured to be in the range of: 5.9–11 μg. A 1:1 ratio of RNAClean xp beads was used to clean up the sample. It was eluted in 9 μL nuclease-free water and directly used for library preparation with the Oxford Nanopore protocol SQK-RNA002. All steps were followed according to the manufacturer's specifications, with increased incubation periods (RNA elution to 15 minutes and adapter ligation to 20 min). The RMX volume was reduced to 4 μl. The final library concentration was in the range of 1044 to 1372 ng. The library was then loaded on an R9.4 flow cell and sequenced on a MinION device (Oxford Nanopore Technologies). The sequencing run was terminated after 72 h. The datasets generated and analyzed for the long-read dataset are available on GEO as a subseries of our main accession (GSE233837) with the accession number: GSE233836.

### Oxford nanopore technology (ONT) data quality and alignment

Long read sequencing was performed using MiniON Initially the ‘fastq’ files were assessed for quality using the command line tool NanoStat (73). Second, the ‘fastq’ files were mapped to the reference mouse genome (GRCm39.103) using the ‘minimap2’ tool (74) and quantified using feature counts from subread package (71) for obtaining gene-level counts. Not that for transcript-level read counts, the files were mapped to the transcriptome ‘minimap2’ tool (74) and quantified using salmon v0.10.2 (72). Third, gene and isoform reads were filtered for low counts (≤5 reads), and quantile normalization followed by a ‘row-wise’ median normalization was performed for ease of further analysis as in the case of the short-read datasets.

### Differential expression and gene ontology analysis

The normalized counts were used to perform Differential gene expression (DGE) analysis using the DESeq2 v1.30.1 (75) R package. Significantly differentially expressed genes (Supplementary Table S2) and isoforms (Supplementary Table S9), with corresponding adjusted *P*-values (*padj*) of ≤ 0.05 and |log_2_FC| of ≥0.58 were selected to determine the functional significance by performing a gene ontology analysis using the clusterProfiler v4.5.0 (76) package. Gene over-representation analysis was performed using the enrichGO() function and gene set enrichment analysis was performed using gseGO() with default parameters and a *p*-value cutoff ≤0.05 and the *P*-values were adjusted using the Benjamini–Hochberg method. Furthermore, the background genes for the over-representation analysis were the genes that were significantly differentially expressed.

### Weighted correlation network analysis (WGCNA) and their association with the cell types and brain pathologies

To construct co-expression networks capturing potentially coregulated genes in the aged brain, we utilized gene-level counts. Initially, the counts were filtered for low counts (≤5 reads), and then we applied quantile normalization, followed by row-wise median normalization to account for differences per gene in the aged brain. The resulting normalized gene-level counts were used as input to identify functional groups using the WGCNA v1.7.1 (77) R package. Gene expression modules or functional groups within a signed network were generated based on the topological overlap matrix (TOM), employing a soft-threshold power of 18 to approximate a scale-free topology. We set the minimum size of the modules at 300 and cutHeight at 0.85. Then the genes were clustered based on the dissimilarity measure (1-TOM). To assess the functional significance of these modules, we performed gene over-representation analysis using the enrichGO() function in the clusterProfiler v4.5.0 (76) R package. The background genes used for this over-representation analysis were all the genes assigned to any of the modules in the WGCNA analysis, ensuring a comprehensive evaluation of functional enrichment. Additionally, we investigated the association of these modules with brain pathologies by analyzing curated and available disease datasets from the literature. The list of the brain pathologies considered for the analysis included Parkinson disease (PD, *N* = 229), mental retardation (MR, *N* = 457), autism (*N* = 924), ataxia (*N* = 245), amyotrophic lateral sclerosis (ALS, *N* = 50), and Alzheimer disease (AD, *N* = 82)). The overlap ratio was calculated using the GeneOverlap v1.26.0 (78) package within R. Furthermore, we explored the association of these modules with specific cell types using the *Tabula muris senis* dataset (6)⁠ (Supplementary Table S7), providing insights into potential cellular mechanisms underlying brain aging. Furthermore, we validated these functional groups or modules using the modulePreservation() function from the WGCNA package. This validation was conducted using a second short-read dataset (50 bp) acquired in an independent cohort of mice, a long-read dataset, and a previously-published human brain aging dataset (7,79) (GSE36192). This comprehensive validation allowed us to assess the preservation and robustness of the identified functional modules across different datasets, enhancing the reliability of our findings.

### GC3/AU3 analysis

For the calculation of GC3/AU3 content, which represents the percentage of the third letter in each codon, we initially selected all genes with isoforms that exhibited significant bidirectional expression changes (both up- and down-regulated). Next, we determined the average GC3 content for each isoform sequence within these selected genes and then computed the mean percentage of GC3 for all isoforms per gene. Subsequently, we categorized the isoforms into two groups based on their direction of expression change during aging (either upregulated or downregulated). For each group, we calculated the difference between the averaged GC3 content of each gene. We then normalized this difference by dividing it by the same value, resulting in a normalized GC3 content value for each isoform group (either upregulated or downregulated). For instance, if there is no difference between the groups, the value reported is 0. Conversely, if there is a difference, the value is either positive or negative, indicating the direction of the change.

### Differential transcript usage (DTU) analysis

To delve deeper into isoform resolution, with a focus on isoform switching and its potential consequences associated with alternative splicing, we performed DTU (Differential Transcript Usage) analysis using the IsoformSwitchAnalyzeR v1.12.0 (80) package. In this analysis, we applied several filtering steps to ensure robust results. Initially, we filtered transcript counts based on geneExpressionCutoff ≤5 and isoformExpressionCutoff ≤3, and only considering genes with at least two isoforms by setting removeSingleIsoformGenes = TRUE. The isoform switches, indicating differences in alternative splicing events, were then identified using the isoformSwitchAnalysisPart1() function, employing the DEXSeq switchTestMethod. To predict the functional consequences of the isoform switches, we employed the isoformSwitchAnalysisPart2() function, considering inputs from CPC2 and PFAM results. We evaluated several consequences, including ‘intron_retention’, ‘coding_potential’, ‘ORF_seq_similarity’, ‘NMD_status’, and ‘domains_identified’. The consequence summary and enrichment were generated using the extractConsequenceSummary() and extractConsequenceEnrichment() functions. The significance test was performed using R’s exact binomial test ‘binom.test()’ with default parameters and the resulting p-values are adjusted with ‘p.adjust()’ using FDR (Benjamini-Hochberg). By performing this comprehensive isoform-level analysis, we gain valuable insights into the intricate dynamics of alternative splicing and isoform switching in the context of brain aging, providing a deeper understanding of the potential functional implications of these molecular events.

### RNA binding proteins (RBPs) and splicing factor (SF) analysis

A list of RBPs and SFs was curated from the RBPmap database (81) ⁠(Supplementary Table S12). The binding motifs of RBPs and SFs were used to determine the binding probability on mRNAs (whole or sub-portions such as the 3′UTR). Initially we calculated the number of occurrences of each motif within the sequence and we then expressed them as binding probability (calculated per 10′000bp). In the case of RBPs or SFs with more than one motif, the probabilities were summed. For the 3′UTR we calculated the fold change (FC) difference between the 24m and 6m by grouping transcripts that are either downregulated, upregulated, or non-significantly changed in the aged brain. In the heatmap (Supplementary Figure S12) the FC was plotted as log_2_, so positive values indicate a relatively higher binding probability in the selected group of genes and *padj* were calculated with ANOVA followed by Tukey post-hoc test accordingly. All the values are in any case reported in Supplementary Table S12 (sheet name ‘RNA binding protein analysis for 3UTR’).

### Comparison to other available published datasets

We compared our data with a human dataset (7,79) (GSE36192). Here a sub-selection of the biological samples from GSE36192 was performed to compare the age group of mice and human as follows: 6m mice were compared to human male cortex samples between the age of 19 and 39 (young adult) and 24m mice were compared to human samples between the age of 48 and 60 (aged). We used the counts deposited in GSE36192, row-median normalized them, and checked for preservation with the modules that we identified in mice through our WGCNA analysis.

To compare the whole brain sequencing data obtained in this study with single-cell datasets, several datasets were used. Initially, since we wanted to check if there a change in the specificity of the expression of neuronal genes is an effect at the level of the whole brain, we used the *Tabula muris senis* database (6)⁠. This database does contain single-cell data for the whole organ and is thus most suited for this purpose. Here we retrieved the counts for various cell types, including non-myeloid cells (neurons, *N* = 14 373; neuronal stem cell, *N* = 1318; astrocytes, *N* = 9181) and myeloid cells (microglia, *N* = 18 052) (6). We normalized the data, and checked the expression of the genes across different cell populations for neuronal genes that were selected from the SynGo Database (82) or for all other remaining genes (Supplementary Figure S2c-f).

To determine if there is a specific cell composition change in the aging brain, we retrieved and reanalyzed datasets from Allen and collaborators (37) and from Buckley and collaborators (39) and performed deconvolution-like analyses to estimate the effect from single-cell populations (83). The dataset from (37) includes different cell types at different ages in the mouse brain. To analyze cell composition, we identified the number of cells per cell type across different ages for excitatory neurons (ExN), inhibitory neurons (InN), medium spiny neurons (MSN), oligodendrocytes (Oligo), astrocytes (Astro), endothelial cells (Endo), microglia (Micro), oligodendrocyte precursor cells (OPC), pericytes (Peri) and vascular leptomeningeal cells (VLMC). We calculated the total number of cells per biological sample and the relative percentage changes of the number of cells across ages. To select genes specific for each cell type, we retrieved their gene counts and calculated the fold change differences across all cell types. When determining cell-specificity for a defined cell type, the genes with a fold change >4 in the remaining cell types were considered specific. After the sub-selection of cell-type specific genes, it was found that macrophages (Macro) had only one gene and T cells did not have any genes and therefore were not included in further analyses. We checked the expression of the cell-type specific genes in the young and aged brain (both in their dataset and in our short-read dataset). The dataset from (39) also includes different cell types at different ages. Since biological replicates are scattered across ages, to be able to obtain mouse groups for direct comparison to our short-read dataset we grouped the ages from 4.7m to 8.4m (average ∼6m) and considered them as ‘young adult’ and from 22.6m to 25.93 (average 24m) and considered them as ‘aged’. To analyze cell composition, we identified the number of cells per cell type for activated neuronal stem cells (aNSC), neurons, neuroblasts, Oligo, Astro, Endo, Micro, OPC, mural, ependymal cells (Epen), and Macro per age group. Further analyses were carried on similarly to what is described above, although a slightly higher threshold of 5 was used as the number of genes was slightly higher. All these results are detailed in Supplementary Table S4, sheet ‘Buckley and collaborators Single-cell’.

To determine if there is a region-specific contribution to the changes that we observed in our short-read dataset we considered the region-specific information contained in the work from Allen *et al.* (37). To identify genes unique to specific brain areas, we collected gene counts for the following distinct regions: upper cortical layers, cortical layers V and VI, striatum, corpus callosum, and olfactory bulb region. We assigned each gene to a single region based on its count compared to the median count in other regions. If a gene's count exceeded the median count of that gene in other areas, it was allocated to that particular region. However, if a gene showed higher counts in multiple regions compared to the median value, it was not assigned to any region. For example, if ‘gene X’ had 100 reads in cortical layer V and this count surpassed the median count for all other brain regions, ‘gene X’ was placed in cortical layer V. Conversely, if ‘gene X’ had higher counts in multiple regions exceeding the median values, it was not considered for assignment to any specific region. To assess the conservation of region-specific information in humans we analyzed data from the Human Protein Atlas (84). Here, we considered genes specific for the cerebral cortex, cerebellum, basal ganglia, thalamus and hippocampus, and the region-specific assignation was directly obtained from that work (84).

For the analysis of CLP1, an important enzyme that can phosphorylate the 5′-hydroxyl groups of RNAs, we selected referred to a previously published work (85). Here we retrieved the genes that are significantly either down- or up-regulated in the CLP1 KO mice versus WT mice (log_2_FC ≥ |0.58| and *padj ≤*0.05) and used these genes for the analysis showed in Supplementary Figure S11f.

### External dataset retrieval information

Data for Supplementary Figure S2 for the single-cell dataset was obtained from cellxgene for Brain myeloid and non-myeloid cells (https://cellxgene.cziscience.com/datasets) (6). Data for Supplementary Figure S1g (human dataset) (7,79) were obtained from Gene expression omnibus (GEO) using the identifier GSE36192. Data for Supplementary Figure S3 and S4 (single-cell and region specific) were obtained from zenodo (https://zenodo.org/records/7145399) (37) and cellxgene (https://cellxgene.cziscience.com/collections/31937775-0602-4e52-a799-b6acdd2bac2e) (39). Data for Supplementary Figure S5 for the region-specific dataset was obtained from human protein atlas (https://www.proteinatlas.org/humanproteome/brain) (84).

### RNA dynamics

To find the RNA dynamics between young adult (6m) and aged mice (24m), we profiled premature and mature RNA expression levels from total RNA-seq data, and we used these quantities to estimate the ratio of processing to degradation rates (i.e. post-transcriptional ratio). A difference of this measure between two conditions is indicative of a peculiar modulation of RNA processing and/or stability (86). Then, we performed a gene-level analysis with the R package INSPEcT (87) to identify the genes that are post-transcriptionally regulated between the two conditions (Supplementary Table S13).

### Plots and statistics

All plots were created in R. Statistical calculations were performed in R as indicated in the text.

## Results

### Gene expression changes in the aged brain

**Figure 1.**
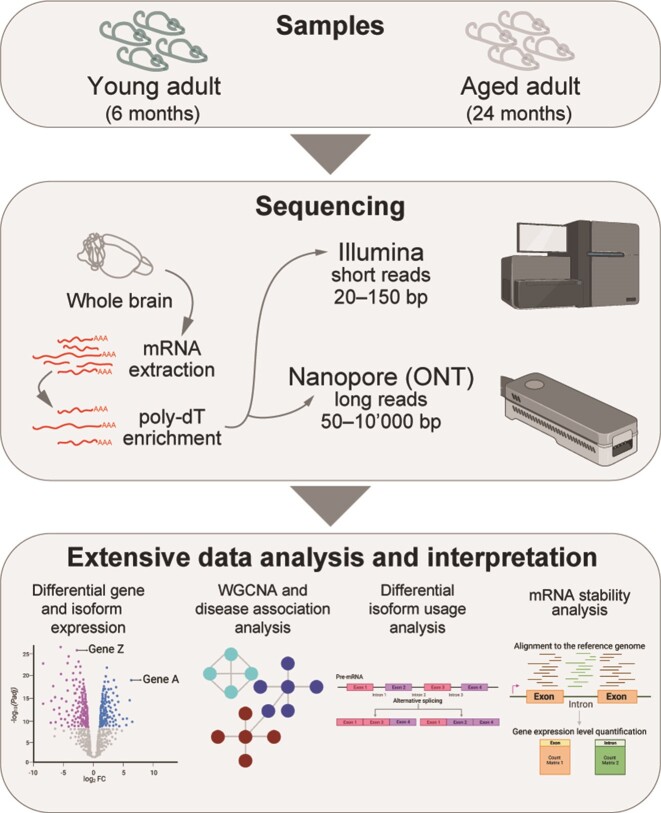
Overview of the experimental outline and analysis workflow.

**Figure 2.**
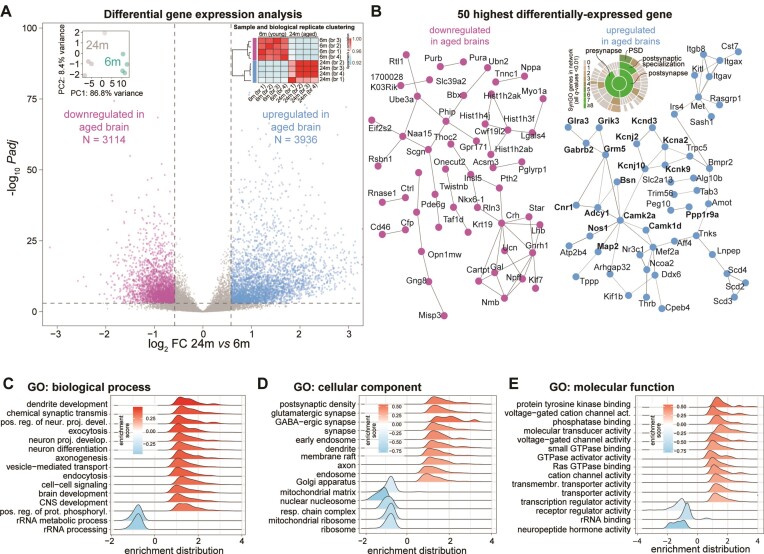
Gene expression changes in the aging mouse brain. (**A**) Volcano plot displaying the differentially expressed genes. Insets: dot plot for biological replicate variability calculated using PCA and correlation matrix (spearman ρ) of the Illumina short-read dataset. (**B**) STRING representation for the top 50 most down- or upregulated genes in the aged brain (respectively pink and light blue). Genes in bold were also identified in a specific synaptic gene ontology enrichment analysis (SynGO (82)), as also shown in the detailing inset where synaptic genes are categorized. (**C–E**) Gene ontology (GO)- Gene set enrichment analysis (GSEA) of genes that are significantly down- or upregulated in the aged brain (*padj* ≤ 0.05, |log_2_FC| ≥ 0.58), related to panel A.

We followed the general experimental workflow explained in the methodological overview (Figure 1) and initially assessed inter-sample variability of the replicates using principal component analysis (PCA) and calculating the Spearman ρ correlation (Figure 2A insets and Supplementary Figure S1a, b, inset). As anticipated, our results demonstrate that the replicates exhibit grouping patterns based on their respective ages. Subsequently, we performed differential gene expression in the aging brain by comparing mRNA abundance obtained through short-read and ONT sequencing methods.

We evaluated genes that exhibited significant differences between 24-month-old (24m) and 6-month-old (6m) mice, selecting genes in our short-read dataset with a multiple comparison adjusted p-value (*padj*) ≤ 0.05 and an absolute log_2_fold change (|log_2_FC|) ≥ 0.58 (Supplementary Table S2). When considering this subgroup of significantly differentially expressed mRNAs, we found a slight over-representation of upregulated genes (3936 upregulated transcripts corresponding to ∼56% and 3114 downregulated transcripts corresponding to ∼44%; Figure 2A, and Supplementary Table S2). We checked and this small bias seems to be due to a *bona-fide* higher expression rather than a problem of normalization. As expected, in the long-read dataset we observed similar changes with an overall significant positive correlation of the two datasets (spearman ρ = 0.74, *P*-value < 2.2e-16; Supplementary Figure S1d and Supplementary Table S2) and also in the second independent animal cohort we observed similar changes with an overall significant positive correlation of the two datasets (Spearman ρ = 0.88, *P*-value < 2.2e-16; Supplementary Table S2).

To check the overall quality of our data, we first made sure that we could find in our data coarse changes consistent with previous aging studies and we could replicate increased expression of astrocyte-specific genes (88–90), including vimentin (Vim) and the astrocyte marker GFAP. These results underscore astrocytosis as a common phenotype of the aging brain (91). Furthermore, we observed the downregulation of sirtuin (Sirt6), a key regulator of mitochondrial function, which indicates mitochondrial impairment (92). We also confirmed the decrease of several other genes for mitochondrially-targeted proteins which are incorporated in mitochondrial complexes such as Ndufs4, Ndufs5, Ndufs6, Sdhb, Uqcr10, Uqcrc2, Uqcrq, Uqcrqb, Cox5a and Cox5b, that corroborate previous observations of mitochondrial dysfunction (93).

When analyzing the highest and lowest 50 most significantly changed transcripts (Figure 2B and Supplementary Table S2), we found that among the differentially downregulated genes in the aged brain several hits indicate changes in neuropeptide hormone secretion and function. These include for example the mRNA encoding Cartpt, an anorectic peptide that inhibits feeding behavior (94), and the homeobox transcription factor Nkx-6–1, which is involved in the transcriptional regulation of the insulin gene and is an important regulator of late brain development (95). Other interesting hits include the ubiquitin protein ligase E3A (Ube3a) and secretagogin (Scgn). Ube3a is an E3 ubiquitin-protein ligase important for the degradation of cytoplasmic misfolded proteins that is mutated in Angelman syndrome, a human neurodevelopmental disorder caused by loss of function of the maternally inherited UBE3A (96,97). Scgn is a secreted calcium sensor that has a function related to neuroendocrine cells and has recently been shown to interfere with α-synuclein fibrillation (98).

Among the 50 most upregulated mRNAs, we observed a clear enrichment of transcripts encoding proteins with essential neuronal and synaptic functions (Figure 2B, right and Supplementary Table S2). These included, for example, mRNAs related to potassium channels (Kcna2, Kcnd3, Kcnj2, Kcnj10 and Kcnk9), neurotransmitter receptors (Grik3, Gabrb2, Grm5, and Cnr1), and other neuronal components, including the presynaptic scaffold bassoon (Bsn) and microtubule-associated protein 2 (Map2). To distinguish possible differences between pre- and postsynaptic changes, we performed an additional analysis using the curated synaptic gene ontology database SynGO (82). This showed that both pre- and postsynaptic mRNAs are increased in the aged brain, although we found a hint among these for the enrichment of components in the postsynaptic density (PSD; inset in Figure 2B). To account for possible sex-chromosome related effects, we also tested if there is a change in the expression of X- or Y-chromosome genes. We observed no difference in their overall expression as they were not significantly changed when comparing 24m vs 6m mice (paired t-test p-values for X-chromosome or Y-chromosome specific gene expression 0.98 and 0.52, respectively).

A more wide-ranging gene ontology gene set enrichment analysis (GSEA) based on all changed transcripts confirmed a significant downregulation of neuropeptide hormone activity (GO:0005184). This analysis also revealed a decreased expression of mitochondrial and ribosomal mRNAs in the aged brain including several gene ontologies (GOs) associated to these processes (e.g. GO:0033108, GO:0007005, GO:0140053 and GO:0032543; Figure 2C–E). Overall, the upregulated GOs point to synaptic transmission, neuronal terminal differentiation, and specific increase of mRNAs for synaptic components (GO:0014069, GO:0098982).

Potential discrepancies in the magnitude and direction of synaptic transcript changes with previous datasets may be due to several reasons, including differences in the age of the control cohorts (refer to Supplementary Table S3) or the fact that subregional studies could suffer from variability in the dissected region. Coincidentally, the latter was one of the reasons that in our case led us to analyze the whole brain. We reasoned that cross-testing our results with recent single-cell datasets could partially resolve some of these discrepancies. In addition, this would allow us to understand whether the observed change is due either to a *bona fide* upregulation of neuronal transcripts in neurons or rather to a deregulation of transcriptional specificity in other cell types, including glial cells, which might begin to express neuronal genes. This possibility is suggested by shifts in the molecular identities of glial cells observed in aged human brains (10). To this end, we took advantage of data from recently published single-cell dataset across mouse ages (6) and we performed a comparative analysis between our datasets and single-cell data (Supplementary Figure S2 and Supplementary Table S4). Due to the increased expression of synaptic genes observed in the aged brain, we focused our analysis on neuronal synaptic genes curated from the SynGO (82) database and examined their expression changes across cell subtypes identified in the single-cell dataset. This analysis revealed that the upregulation of synaptic genes is significant in aged mouse neurons but was not observed in other cell types (Supplementary Figure S2). Overall, these findings indicate a possible engagement of neurons to produce more synaptic transcripts in the aged brain.

To investigate whether the composition of cell types and specific cell populations might influence some of our results, we additionally utilized two recently published single-cell datasets (37,39) covering various mouse ages and applied single-cell deconvolution-like approaches inspired by the recent literature (83). Re-analysis of these single-cell datasets allowed us, first, to answer the question of whether changes in the number of different single-cell populations during brain aging were clearly detected in these single-cell studies. Second, we also used these data to test, at least by estimation, whether the differences observed in our work might be preferentially due to specific cell populations. After retrieving and re-analyzing the data, we found no significant differences in cell numbers across ages in the single-cell datasets (Supplementary Figure S3a, b), with two exceptions: microglial cells in the work of Buckley and coworkers ((39), Supplementary Figure S3a) and endothelial cells in the work of Allen and coworkers ((37), Supplementary Figure S3b). We decided to follow up on the observation that microglia cells are increased in the work from Buckley and collaborators. After retrieving the list of 1330 microglia-specific transcripts from their work, we checked if these genes are changed across ages in our dataset (Supplementary Figure S3c). With this analysis we found that microglial genes are indeed also increased in our aged dataset. To understand if this phenotype could also be due to a change in the expression profile of this cell population, we performed GO analysis of the microglial-specific genes (Supplementary Figure S3d) and found an overrepresentation of terms related to microglia activation such as lytic vacuole (GO:0000323) and phagocytic cup (GO:0001891), suggesting a possible change in both number and phenotype of these cells.

We conducted a similar GO analysis on the endothelial cells (Supplementary Figure S3e), but no significantly enriched terms were found, suggesting no change in the phenotype of these cells.

To additionally explore whether there is a possible influence of region-specific changes in our bulk transcriptome data, we re-analyzed the region-specific data from Allen and colleagues (37). For each brain region, here we defined genes that could serve as region-specific markers and checked if they were changed in our aged short-read dataset (Supplementary Figure S4a). This analysis showed a significant upregulation of genes enriched in cortical layer V, in the striatum, and in the corpus callosum during aging. Moreover, to extend our findings to the human context, we also selected the region-specific genes from the Human Protein Atlas (84). Despite overall module conservation (Supplementary Figure S1g), we found no significant differences during aging in our dataset when analyzing region-specific genes defined in human (Supplementary Table S3; Supplementary Figure S5), suggesting that region-specific differences might be less conserved between species.

### Identification of common gene expression modules during brain aging

**Figure 3.**
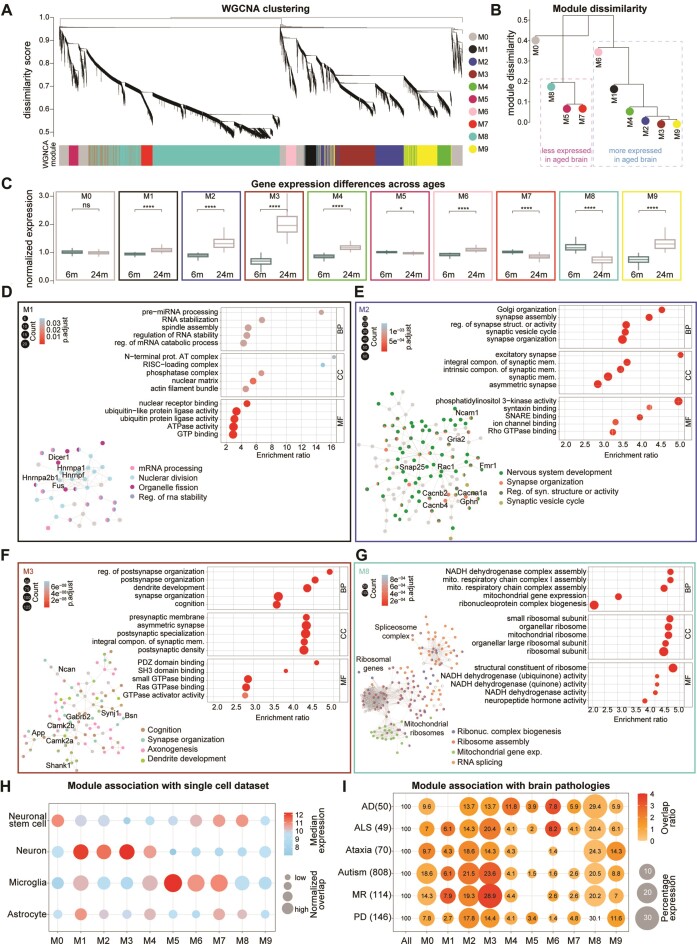
Co-expression modules and association with cells and brain pathologies of the mouse aging transcriptome (**A**) WGNCA dendrogram with highlighted modules (lower colored bar). Genes were clustered based on a dissimilarity measure. The branches are modules of closely correlated gene groups. Nine significant modules and M0 corresponding to ∼16 000 genes were detected with WGCNA. M0 is a module with a less correlated gene group. (**B**) Module dissimilarity based on module eigengene distances. Modules are grouped based on their expression. (**C**) Boxplot of normalized mRNA expression in young and aged brains, grouped by their modules detected in the WGNCA method (paired t-test followed by Tukey posthoc test *P*-value * ≤ 0.05, ** ≤ 0.01, *** ≤ 0.001 and **** ≤ 0.0001). (**D**–**G**) Gene ontology over-representation analysis (GO-ORA) for the four selected modules, respectively M1, M2, M3 and M8 shown here are top 5 GO terms based on the enrichment ratio. For all modules in detail refer to Supplementary Table S6. Circle sizes in the enrichment graphs correspond to the number of terms for each GO term, and color scales represent the *padj*. Insets are string network analysis for each module and their association with the respective pathways. For enlarged views of the string network analysis refer to Supplementary Figures S6–S9. (**H**) Dot plot of median mRNA expression from a previously published cell dataset. The size of the bubble represents the normalized overlap of the genes in the module with the specific cell type Supplementary Table S7). The color scale corresponds to the median expression for the overlapping genes. (**I**) Dot plot of the association of modules to brain pathologies. Lists have been manually curated for each pathology (Supplementary Table S8), the numbers are for overlap with all the genes in the short-read dataset, Alzheimer's disease (AD), amyotrophic lateral sclerosis (ALS), ataxia, autism, mental retardation (MR), and Parkinson disease (PD). The size of the bubble represents the log_10_*P*-value, and the color scale corresponds to the overlap ratio. The numbers in the bubble are the percentage of genes in each module for each pathology.

To gain a more precise molecular understanding of the transcriptome changes and to identify patterns of gene expression and relationships between genes during aging, we examined gene co-regulation using weighted gene co-expression network analysis (WGCNA (77)). This method allows to cluster genes with similar expression patterns into modules and can provide clues to potential regulatory relationships between genes while reducing the complexity of the data. Using this approach, we identified nine mutually exclusive significantly different modules of different sizes ranging from 417 to 6878 genes (Figure 3A–C and Supplementary Table S5), and one module (M0) corresponding to genes that remain unassigned. Among the nine modules in the aged brain six were upregulated (M1, M2, M3, M4, M6 and M9) and three were downregulated (M5, M7, and M8) (Figure 3A, B). Note that the clustering method does not take age group information into account, and the fact that we found several modules that were differentially expressed across ages indicates the ability of our method to identify gene expression patterns that are relevant to the aging process.

To obtain a biologically-oriented description of each module, we performed an over-representation analysis using GO annotations (Supplementary Table S6). Several significantly enriched biological processes were identified for each module (Supplementary Table S6, Figure 3D–G, Supplementary Figure S1f and Supplementary Figures S6–S9), allowing to identify modules related to neuronal and synaptic biology (M2, M3, M4, M9), RNA stabilization and processing (M1), mitochondrial and ribosomal function (M8), and immune response where two modules (M5, M6) displayed opposite expression patterns (Figure 3C, Supplementary Figure S1f). Although the data for all modules are available (Supplementary Table S6), due to space limitations, we focused on the detailed description of four modules that we found to be of particular interest: (i) M1 which is overexpressed in the aged brain, encodes a number of proteins important for regulating mRNA stabilization and processing including Dicer1 that orchestrate short dsRNA-mediated post-transcriptional gene silencing and FUS, an RNA-binding protein that plays a pivotal role in RNA metabolism (Figure 3D and Supplementary Figure S6); (ii) M2, which is also increased in the aged brain and encodes several components important in the development and the final differentiation of neurons and particularly pre-synapses. Among these there are SNARE proteins such as SNAP-25, the RNA-binding protein FMRP (encoded by Fmr1) and important synaptic adhesion molecules, such as the axonal neural cell adhesion molecule 1 (Ncam1; Figure 3E and Supplementary Figure S7); (iii) M3, also an aged-overexpressed synaptic module, is predominantly enriched for transcripts encoded proteins related to cognition and synaptic scaffolds.

For example, it includes the adapter protein of the excitatory postsynaptic density Shank1, and different isoforms of the Calcium-calmodulin kinase II (Camk2a and b) that are localized at the postsynapse (Figure 3f and Supplementary Figure S8); (iv) M8, which is downregulated in the aged brain, includes a large number of ribosomal genes, both cellular and mitochondrial and a series of mRNA encoding important players in RNA splicing, such as the core component of the spliceosome (Figure 3G and Supplementary Figure S9).

To further explore the association of the identified modules with different brain cell populations and to explore possible links to human brain pathology, we used the previously mentioned single-cell dataset (6) (Figure 3H; Supplementary Table S7) and we curated a set of genes related to human pathology (Figure 3I; Supplementary Table S8). These analyses showed that the modules M1-M4 have a specific association with the neuronal cell type and a higher overlap with brain pathologies. Modules M5-M6 are associated with microglia and a comparatively lower association with brain pathologies (Figure 3I). Moreover, we checked if the region-specific genes identified by Allen and colleagues (37) showed age-related differences in our modules. We concentrated on the gene sets which we found significantly different in our dataset (cortical layer V, striatum, and corpus callosum; Supplementary Figure S4a). This analysis revealed that cortical layer V genes are significantly increased in aged brains in our module M3 (related to synaptic neuronal function; Supplementary Figure S4b). When considering striatum-specific genes we found no significant difference across our modules (Supplementary Figure S4c). Finally, the corpus callosum-specific genes were increased in aged brain in modules M4 and M9 (respectively a synaptic module and a module related to cellular response to lipid and small GTPase function; Supplementary Figure S4d).

### Age-related modules are conserved in independent human brain and mouse datasets

As a complementary approach to validate and further extend the relevance of our findings to the human context, we performed module preservation analysis using both of our datasets (short and long read) and a publicly available human dataset (7,79). For the analysis of the human dataset, we defined the young adult group as individuals aged between 20 and 30 years of age and the elderly group as individuals between 56 and 69 years of age. We selected these age groups as they are more adequate for comparison with mouse brains (99) This analysis revealed that modules M1 to M4, which were found to be upregulated in the aged brain in our short-read pair-end mouse dataset, exhibited preservation in a second independent animal cohort (Supplementary Figure S1f), upon sequencing with a different technology that has inherently different technical biases (Supplementary Figure S1e; (100,101)), and also in the human context (Supplementary Figure S1g). This preservation across species and datasets confirms the robustness of our observations and indicates that the upregulation of genes related to neuronal function is well conserved and is also present in humans, when adult mice are used for the comparisons.

### Altered splicing and length-associated transcriptome differences in the aged brain

Given the abundance of alternative splicing (AS) events in the brain (43), and the finding that a module we identified directly points to changes in mRNA splicing (M8), we investigated more specifically whether there are age-specific changes in AS. For this purpose, we initially focused on our short-read dataset, aligning it with the entire mouse transcriptome and we quantified distinct alternative isoforms expressed for each gene.

For further analyses we concentrated on genes that have at least 2 isoforms that exhibited significant differences between 24m and 6m mice (Supplementary Table S9, S10). In our short-read dataset, we found that ∼53% isoforms are upregulated in the aged brain (*N* = 6984) and ∼46% are downregulated (*N* = 6091; Supplementary Table S9), in line with what we have seen at the whole-gene level. In the long-read dataset we observed very similar changes for isoforms that were identified in both the technologies with a significant positive correlation between the two datasets (spearman ρ = 0.46, *P*-value < 2.2e-16; Supplementary Table S9). In the second independent animal cohort dataset also, we observed very similar changes for isoforms that were identified in both the cohorts with a significant positive correlation between the two datasets (spearman ρ = 0.87, *P*-value < 2.2e-16; Supplementary Table S9). In addition, we examined whether there was a difference in the number of splicing isoforms detected in either the young adult or aged brain after applying a minimal cutoff. The analysis showed that there is indeed a slight increase in the number of spliced isoforms in the aged brain (on average 3.3% more isoforms in the aged brain).

**Figure 4.**
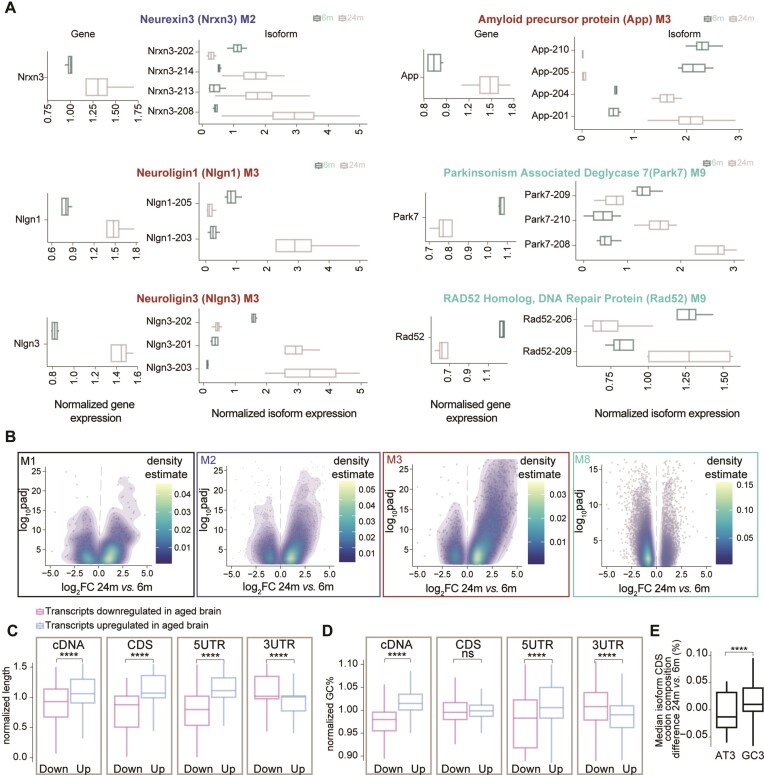
Isoform expression changes in the aging brain. (**A**) Boxplot of the normalized expression for a subselection of genes having isoforms that are expressed in an opposite manner when compared to the respective module. The panel on the left shows the gene expression while the right panels isoform expression. (**B**) Volcano density plots of significantly differentially expressed mRNA isoforms within selected modules from the WGCNA analysis that show interesting patterns during brain aging (M1, M2, M3 and M8). (C, D) Boxplot of the length (**C**) and GC% for different features (**D**) of an isoform. Genes were selected if they have at least 2 isoforms differentially expressed (*padj* ≤ 0.05 and |log_2_FC| ≥ |0.58|). Numbers were normalized for each gene and values of 1 would indicate equal distribution between age groups. (**E**) Boxplot of codon composition in the CDS for genes that were selected if they have at least two isoforms differentially expressed (*padj* ≤ 0.05 and |log_2_FC| ≥ |0.58|; paired t-test followed by Tukey posthoc test * ≤ 0.05, ** ≤ 0.01, *** ≤ 0.001 and **** ≤ 0.0001)

Some notable examples of AS changes were observed for known adhesion molecules linked to synaptic function and formation/maintenance (102), such as Neurexin 3 (M1), Neuroligin1 and 2 (M3). These three proteins, when considered at the gene level, all follow the expression trend of their respective modules and are overexpressed in the aged brain (Figure 4A, respective panels on the left). When annotating the sequencing results, taking into account the different isoforms derived from the same gene, we realized that each of these genes also had isoforms that were expressed in the opposite manner to their respective modules (in these cases, downregulated during aging), such as an example Nlgn1-205 and Nlgn1-203, corresponding to two transcripts which are protein coding but that in the case of Nlgn1-205 goes in the opposite direction than M3 (downregulated in aging while genes in M3 are upregulated in aging, Figure 4a right panels). These observations indicate an isoform switch occurring during aging, which we observed for at least ∼4300 genes. Interestingly, also genes associated with neurodegenerative diseases (NDDs), such as the mRNA encoding for the amyloid precursor protein APP linked to Alzheimer's disease and Parkin-7 (Park7) which is associated with Parkinson's disease (PD) show isoform switching behavior during brain aging. In the case of APP, isoforms App-201 and App-204, which encode the amyloid-β domain associated with AD pathology, were upregulated in the aged brain, consistent with gene expression changes. Conversely, the isoforms App-205 and App2-10, which do not encode a protein, showed lower expression in the aged brain. These findings align with the observation that isoforms with higher expression in the aged brain are predominantly protein-coding (Supplementary Figure S11c). Similarly, for the Park7 gene, isoform Park7-209 was downregulated in the aged brain, mirroring the gene expression pattern, while isoforms Park7-209 and Park7-210 were upregulated. Additionally, we examined the expression of Rad52, a gene involved in DNA repair that exhibits dysfunction during brain aging (71,72⁠). We found that Rad52-206 was downregulated, consistent with gene expression changes, while Rad52-209 was upregulated in the aged brain (Figure 4A). When considered for each module, isoform switching behavior, also referred to as ‘bidirectional expression’ here, was observed for ∼28% of the isoforms (see for example isoform density plots for overexpressed modules in the aged brain such as M1, M2 and M3 and downregulated modules such as M8 in Figure 4B). In the second independent animal cohort dataset, we also observed a similar pattern in the changes of isoform switching that was conserved across modules (Supplementary Figure S10a, b). Furthermore, consistent with the switching pattern results, the analysis of module conservation indicates that modules are being preserved (Supplementary Figure S1f). It is reassuring to note that the modules are conserved even when the results were obtained in two independent animal cohorts.

In the light of this widespread isoform-switching behavior observed in the aged brain, we decided to investigate the potential aspects influencing isoform changes. For this, we assessed whether general features related to mRNAs such as isoform length, mRNA percentage of guanine cytosine (GC%) which correlate to RNA stability, and codon composition could contribute to the switching (Figure 4C, D). To account for a potential bias introduced by isoforms upregulated in the aged brain (which are by definition more numerous), we performed a gene-wise analysis, which is not influenced by expression differences (see methods for details). Briefly, we focused on genes with at least two isoforms expressed in both age groups and normalized the length and the GC%, separately for the isoforms that were significantly upregulated or downregulated in the aged brain within each gene. To have a more precise understanding of which region of the RNA could be specifically influenced by these changes we analyzed both the mRNA as a whole (cDNA) or subdivided it into coding sequence (CDS) and in the 5′ or 3′ untranslated regions (UTRs). This analysis revealed that isoforms overexpressed in the aged brain (up) when considered as a whole (at the level of the cDNA) tend to be longer (Figure 4c leftmost panel). When considering the CDS and the 5′UTR, which collectively account for ∼70% of the cDNA length, this observation was conserved. In contrast, the 3′UTR exhibited a reverse trend (Figure 4C, rightmost panels).

We also found that among the genes with isoforms that have bidirectional expression in the aged brain, those that are overexpressed are significantly longer than those that are downregulated (Supplementary Figure S13a left panel). Furthermore, we observed a positive correlation for module M2 (neuronal and synaptic; spearman ρ = 0.1 and *P* = 2e-05) and M8 (mitochondrial and ribosomal; spearman ρ = 0.29 and *P* = 2.2e-16). We also observed a negative correlation for module M4 (vesicle organization; spearman ρ = –0.12 and *P*-value = 0.00072) and M5 (immune response, spearman ρ = –0.11 and *P*-value = 0.027). Additionally, an overall positive correlation between gene length and expression levels (spearman ρ = 0.2, *P*-value 7.13e-96) (103), consistent with previous observations in the brain, but in contrast to the generally negative correlation reported for other tissues. This could suggest that longer isoforms are subjected to less efficient degradation in the aged brain, leading to their accumulation and increased abundance. When analyzing the GC%, which is known to be correlated to increased mRNA stability (104,105), we observed a very similar trend (Figure 4D), although the CDS was not significantly different between aged and young adult brains. We and others have reported a specific pattern of genes that contain different GC% (99,100). When considering more specifically the G/C-ending codons (GC3), we also observed that aged isoforms have a higher GC content at the wobble nucleotide position (Figure 4e). For both length and GC% a reverse trend was observed for the 3′UTR. This might be of particular importance as the 3′UTR region is known to regulate mRNA dynamics and stability (44), as for example it is a site for miRNA-mediated targeting and degradation.

Collectively, these findings highlight age-specific changes in AS in the brain, evidenced by bidirectional isoform expression of key genes, as well as by the differential expression of isoforms associated with NDDs and DNA repair. Moreover, transcript length, GC% content, and codon composition appear to contribute to the observed isoform changes in the aged brain.

### Age-specific alternative splicing events

To understand if there is a specific pattern in splicing events that could be at the basis of the observed isoform differences, we performed differential transcript usage (DTU) analysis. This analysis allows to differentiate subtypes of splicing events. Through this analysis, we identified specific splicing events for 5325 isoforms corresponding to 2311 genes (Supplementary Table S11). Alternative splicing might be generated by variations in transcription start or termination sites (ATSS, ATTS), 5′ acceptor 3′ donor splice sites (A5, A3), exon skipping (ES), intron retention (IR), multiple exon skipping (MES), and insertion of mutually exclusive exons (MEE), as⁠ also represented in Supplementary Figure S11a. In this type of analysis, 0.5 signifies no difference among the groups that are considered, while lower or higher numbers indicate either a decreased or increased occurrence of the AS event.

**Figure 5.**
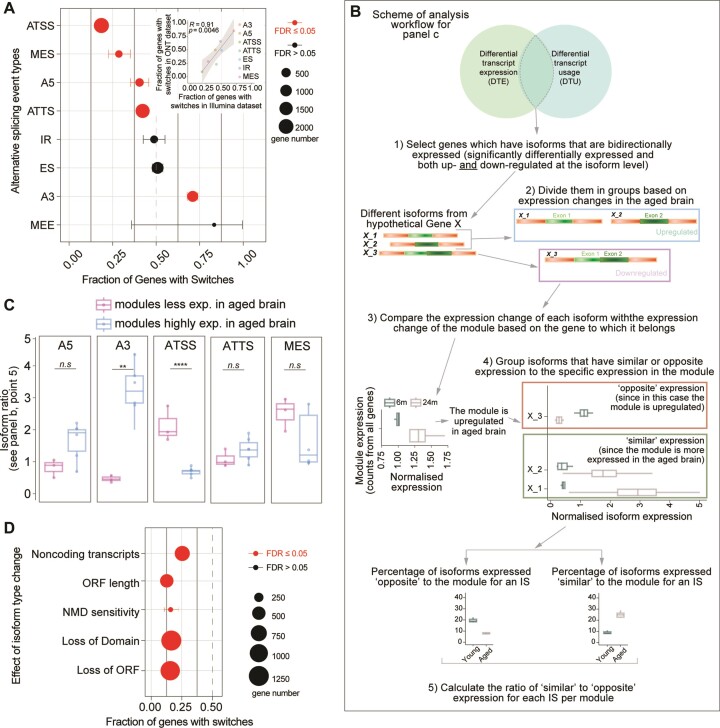
RNA splicing changes in the aging brain. (**A**) Alternative splicing events in the aging brain (FDR ≤ 0.05 are significant changes), inset: scatter plot summarizing the correlations of the short-read with the long-read dataset for each splicing type. Points represent the fraction of genes with switches for each functional category in the individual datasets (Pearson's *P* values). The significance test was performed using an exact binomial test followed by Benjamini-Hochberg adjustment (as this test is more accurate to study a variable that can take only two possible values; * ≤ 0.05, ** ≤ 0.01, *** ≤ 0.001 and **** ≤ 0.0001). (**B**) Workflow to compare the differentially used and expressed transcripts. (**C**) Isoform ratio as calculated in point 5 of panel B in figure. Modules are grouped depending on whether they are less expressed in the aged brain (M5, M7, M8) or more expressed (M1, M2, M3, M4, M6, M9) (unpaired t-test followed by Tukey posthoc test **** ≤ 0.0001). (**d**) Consequences of isoform types in the aging brain (FDR ≤ 0.05 are significant changes). The significance test is performed using the exact binomial test followed by Benjamini–Hochberg adjustment (* ≤ 0.05, ** ≤ 0.01, *** ≤ 0.001 and **** ≤ 0.0001).

Analyzing our dataset, we observed that A5, ATSS, ATTS and MES events had a significantly decreased occurrence in the aged brain (<0.5), while the A3 events were increased (>0.5; Figure 5A). We also considered the same changes in the long-read dataset, and we observed that changes in both datasets were highly correlated (*r^2^* = 0.90, *P* = 0.0046; Figure 5A inset and Supplementary Figure S11b). The use of these two complementary datasets ensured the reliability of the observed changes, although the overall number of isoforms subdivided for each category is in some cases low (<20).

To investigate whether the type of alternative splicing is related to the change in expression during aging, or in other words if an isoform that is either more or less expressed in the aged brain can be influenced by a specific splicing event, we designed a specific analysis (Figure 5B). First, we identified the isoforms that are significantly differentially expressed in the aged brain and that have a different splicing type (differentially used). We then focused on those genes whose isoforms are bidirectionally expressed (e.g. see Figure 4A) and we grouped them based on their module identities as defined by our WGCNA analysis (Figure 3). This allowed us to identify isoforms with either ‘similar’ or ‘opposite’ expression changes when compared to the module affiliation at the level of the whole gene. As an example, the gene App (amyloid beta precursor protein, Figure 4A) belongs to M3 but has four isoforms, two of which are upregulated in the aged brain (App-201 and APP-201) and two which are downregulated (App-205 and App-210) (Supplementary Table S9). Since M3 is upregulated in the aged brain (Figure 3B), App-201 and APP-201, the two upregulated isoforms, have a ‘similar’ expression to M3, while the two downregulated (App-205 and App-210) have an ‘opposite’ expression to the module. We extended this analysis to the selected isoforms as schematized in Figure 5B and we found that modules overexpressed in the aged brain (M1, M2, M3, M4, M6 and M9) when compared to modules that are downregulated in the aged brain (M5, M7 and M8) predominantly contained isoforms with an alternative 3′ acceptor site splicing (A3; Figure 5C). Isoforms showing an ATSS splicing event showed an opposite phenotype (Figure 5C). These findings indicate that A3-type splicing events might stabilize isoforms that are overexpressed in the brain (and thus contribute to their increased levels), while ATSS-type splicing events might have the opposite effect. Additionally, this analysis indicated that in the aged brain the overexpressed isoforms tend to be less sensitive to nonsense-mediated RNA decay (NMD), further corroborating this hypothesis, and have more abundant protein-coding isoforms and less abundant noncoding transcripts (Figure 5D, Supplementary Figure S11c). Collectively, these findings suggest that age-specific isoform switches in the aging brain, might have consequences for mRNA turnover due to reduced NMD sensitivity and possibly other mechanisms that render some mRNAs less efficiently degraded.

To test whether specific mRNA-binding proteins (RBPs) and/or splicing factors (SFs) could explain the differences in splicing events observed in the aged brain, we performed an *in silico* RNA-binding motif analysis, using ∼80 proteins with well-defined binding specificity (72). This analysis revealed at least 7 RBPs that show higher binding probability for isoforms that are significantly altered in the aged brain (Supplementary Figure S11d, e and Supplementary Table S12), including RBM4 (downregulated in the aged brain, Supplementary Table S2), which is associated with neuronal differentiation (44), and HNRNPU (upregulated in the aged brain, Supplementary Table S2), which is related to neuronal development (106). Additionally, we considered the RNA kinase CLP1, as this protein has a role in the 3′-end formation (107) and used a published dataset for retrieving mRNAs that change when CLP1 is knocked down ((85). Supplementary Figure S11f). This analysis indicates that overall genes regulated by CLP1 (independently of whether they are down or up-regulated) tend to be more expressed in the aged brain and suggests that CLP1 might have a role in the regulation of the aged brain transcriptome.

We also performed a specific RNA-binding motif analysis for the 3′UTR region of mRNAs, as this region generally plays a predominant role in mRNA stability and regulation (Supplementary Figure S12). Our results show several significances, although overall there is no clear pattern of RBPs and SFs that could easily explain the differences in mRNA expression that we detect during aging. This is because none of the proteins analyzed shows a significant bidirectional pattern; in fact, if this were the case, we could hypothesize that a specific RBP or SF drives some of the expression differences we observe. Interestingly, we observed that overall, the mRNAs that change their level during brain aging (both increased and decreased) are enriched for RBP and SF binding motifs (46 among the 50 significant for downregulated transcripts and 23 out of 33 significant for the upregulated transcripts). This finding suggests that the 3′UTR region of mRNAs might be particularly sensitive to alterations in RBP and SF networks, although further experiments are needed to verify this experimentally.

### Post-transcriptional mRNA regulation in the aging brain

**Figure 6.**
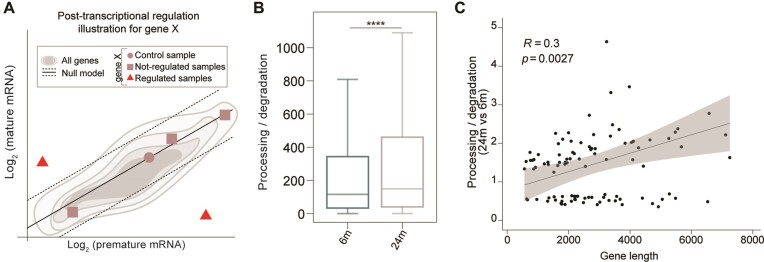
Post-transcriptional mRNA regulations in the aging brain. (**A**) Cartoon illustrating the rationale behind the identification of the samples characterized by a post-transcriptional regulation of a hypothetical gene ‘X’. A linear model is fitted in the log_2_ (premature) – log_2_ (mature) space to recapitulate gene expression levels (gray shadows). The linear model is then translated to interpolate the control sample (dot). This leads to a gene-specific null model (black solid line). The samples not compatible with the null model (red triangles) would be evidence of a post-transcriptional regulation of a hypothetical gene ‘X’ while the other samples do not (squares). (**B**) Post-transcriptional ratio distributions in 6m and 24m mice (18638 expressed genes - Kolmogorov-Smirnov and paired Wilcoxon tests *P*-values **** ≤ 0.0001). (**C**) Pearson's coefficients summarizing the correlations of gene length to the estimated mRNA lifetime changes in the aged brain. Points represent the posttranscriptional ratio and gene length (Pearson's *P*-values).

To follow up on the suggestion that some mRNAs may be less subject to degradation in the aged brain, we examined changes in the ratio of post-transcriptional rates, similarly to what has been done previously(87). Specifically, we used total RNA-seq data to profile premature and mature RNA expression levels, allowing us to estimate the ratio of processing to degradation rates, also known as post-transcriptional (PT) ratio, which is a measure influenced by mRNA turnover. A difference in this measure between the aged and young adult brain indicates potential modulation of RNA processing and/or stability (Figure 6A and Supplementary Table S13). Genome-wide analysis revealed a significant increase in PT ratio in the aged brain compared to the young adult brain (Figure 6B—Kolmogorov–Smirnov and paired Wilcoxon tests *P*-values = 0), suggesting a higher processing rate and/or increased RNA stability in the aged brain.

To gain a detailed understanding of the genes that contribute to the differential phenotype observed in the aged brain, we performed gene-level analysis using the R package INSPEcT (86,87). This analysis led to the identification of 125 genes that were post-transcriptionally regulated between the two conditions, a number significantly higher than expected from random models (Supplementary Figure S13b). Strikingly, this set of genes was enriched in transcriptional units involved in RNA metabolism and immune cell defense (Supplementary Figure S13c), indicating the potential involvement of mRNA turnover in shaping specific biological processes that are hallmarks of brain aging. Finally, we further explored the relationship between PT and gene length and observed a positive correlation (Figure 6C) and found that these transcripts are significantly longer in the aged brain (Supplementary Figure S13a, left panel).

## Discussion

The aging brain undergoes a multitude of molecular changes that influence its function and susceptibility to age-related neurodegenerative disorders. In this study, we have undertaken a comprehensive characterization of the transcriptome in the whole aging brain combining two sequencing technologies, analyzing a second animal cohort for replication purposes, and applying several detailed bioinformatic analyses that were customized based on our observations. We focused on mRNA level changes, isoform usage, and modulation of post-transcriptional RNA dynamics to shed light on the complex molecular processes underlying brain aging.

Mouse lines can provide genetically homogeneous and disease-free cohorts of animals and are often used for aging studies (108). Several works concerning brain aging changes at the transcriptome level have been published (6,8,9,11–14,16,17,19,79,109) and different results have been found, possibly due to differences in the health state or age of the animals considered (refer to Supplementary Table S3). In both mice and humans, the period of adolescence is characterized by several changes in brain connectivity and synaptic refinement. In humans, this culminates in comprehensive cerebral maturation towards the end of the second decade of life (106), whereas in mice, brain maturation lasts until 6 months of age (110,111). For this reason, in this work, we selected 6-month-old mice as a control group for comparisons with aged mice.

A clear finding of our work is that aged brains show the downregulation of transcripts related to ribosomal and mitochondrial function, accompanied by an upregulation of several mRNAs encoding neuronal transcripts (Figure 2). We also observed a mild astrocytosis, highlighted by the increase of astrocyte-specific genes such as GFAP, similar to previous findings (13,88). We did not observe overt inflammation in our samples, which is somewhat in contrast to previous reports indicating strong immune activity in the aged brain (1,2,112). This difference could be due to the age of the ‘young’ control cohort of mice used for comparison and possibly also to the health status of the aged colony in other works. At the same time, careful reanalysis of single-cell datasets and combination with our results does show an increase in the expression of microglial genes in the aged brain (Supplementary Figure S3c). This apparent discrepancy points to a possible problem in the curation of the current GO terms, which will need to be updated in the light of the latest single-cell results. A deeper analysis also suggests caution in the interpretation of these results, as these cells could either increase in number or change their phenotype, since we found evidence of genes related to microglial activation, in agreement with previous results (21,113).

Another interesting finding is that we consistently observed a higher proportion of significantly overexpressed rather than downregulated transcripts in the aged brain (56% in short read and 75% in long read). Although we can only speculate about this finding, it may suggest that either increased mRNA production, decreased degradation, or both processes are occurring in the aged brain. If recent findings obtained in the liver could also be extended to the brain (114), the increased percentages of elongating RNA polymerases would suggest that the mRNA degradation is the most affected part of this dynamic equilibrium. Importantly, among the transcripts that more pronouncedly show this effect there are those encoding for proteins with essential neuronal and synaptic functions (Figure 2B), as also observed in the upregulated modules related to neuronal and synaptic biology (M2, M3, M4 and M9; Figure 3B). When re-analyzing region specific datasets, we also observed an association between the genes of the M3 module and cortical layer V and between the corpus callosum and M4 and M9 (Supplementary Figure S4b, d). This indicates that some of the changes that we observe in our bulk dataset are likely to be more pronounced in specific brain regions.

Regarding the open debate about the possibility that physiological aging might reduce the number of neurons (115–119), our results, while far from definitive, seem to against a net loss, at least in mice. This was also cross-checked with two recent single-cell datasets (37,39), where the only significantly changed cell populations were microglia and endothelial cells (Supplementary Figure S3c, e). We believe that the number of endothelial cells is likely to be increased in the aged brain as both our data (Supplementary Figure S3e) and the work of Allen and coworkers (37) points to this, but also here caution is needed as previous works indicate that endothelial cells in the brain might decline with age (120–122). The study of brain endothelial cells is particularly relevant because recent parabiosis experiments, in which aged mice are exposed to the rejuvenating circulation of younger animals, show specific reprogramming of this subpopulation of cells (123). It is clear that that these cells might be a promising target for the treatment of age-related diseases and should be studied more in detail. More in general, re-analysis of single-cell datasets shows that changes in cell populations during brain aging are either minute or very difficult to be detected even with the latest single-cell approaches (Supplementary Figure S3a, b; (37,39)), and specific enrichment approaches or more detailed experimental strategies will be required to obtain a definitive picture.

We see increased expression of mRNA for synaptic components, suggesting that there is no bulk neuronal loss and possibly even some positive compensation. Moreover, re-analysis of an independent single-cell database (6) indicates that the cell type expressing these mRNAs is indeed neuronal (Supplementary Figure S2), ruling out the possibility that glial cells undergo partial trans-differentiation and begin to express neuronal transcripts not specifically in the aged brain.

Mitochondrial dysfunction is believed to be critical in the onset and progression of neurodegenerative disorders (124,125). We also observed this phenomenon in our dataset (M8, Figure 3I). Additionally, we found that the modules (M1-M3) upregulated in the aged brain are associated with brain pathologies (Figure 3I) and are conserved in a human dataset (Supplementary Figure S1g).

While analyzing our data, we realized that one module (M8) directly points to changes in mRNA splicing and decided to look at splicing in detail. The role of splicing in brain aging has been addressed in part by previous work (28,103), but none of these efforts have attempted to understand whether there are general shifts in splicing behavior in this context. Our work, which serves as a large database of precise splicing changes for mRNAs encoding important processes such as synapse formation, maintenance, and modulation of brain activity (Supplementary Table S6), also allowed us to test for these global changes. The isoform analysis allowed us to confirm that even for the same gene, longer isoforms tend to have higher levels in the aged brain (Figure 4C; Supplementary Figure S13a, left panel) and that this is true when separately considering most regions of the mRNA, including the CDS and the 5′UTR, while the 3′UTR showed an opposite trend. This finding supports our gene-level results suggests that there may be something specific about the brain for the accumulation of longer mRNAs, while other tissues show an opposite trend (96). This point is controversial in the literature as there are indications that support both positive and negative correlation between gene length and increased expression during brain aging (103,114,126). These might be due to differences in the age of the control mice that were used for comparisons (126). At the same time, even a more careful module-wise analysis of our data shows differences in the correlation between isoform length and expression. For example, we observe a significant positive correlation for the neuronal module M2 and the mitochondrial/ribosomal module M8, while the vesicular module M4 and the immune response module M5 show an opposite trend. These contrasting results point to an unknown molecular or evolutionary mechanism that influences the relationship between mRNA length and expression change during aging, precluding a simple interpretation of these correlations.

Other aspects related to RNA composition, including GC%, seem to play a role, indicating either the existence of specific binders, or biophysical stabilization processes that might explain these effects. We attempted to rely on available in silico data for RBPs that could explain some of the splicing changes that we observed and found some potentially interesting regulators. As an example, HNRNPU, KHDRBS2, RBM25, RBM4, DAZP1 and UNK have binding motifs that are overrepresented in the isoforms more expressed in the aged brain (Supplementary Figure S11d, e). The paradoxical result of the 3′UTR may be related to an additional regulatory layer that may play a role here, as miRNAs usually interact with the 3′UTR of target mRNAs. We also observe that both RBPs and SFs overall have a higher binding frequency in mRNA isoforms that are differentially expressed in the aged brain (Supplementary Figure S12). These results remain puzzling and future studies that unbiasedly and comprehensively test the preferential degradation of RNA based on their 3′UTRs. Moreover, a comprehensive analysis of the RBPs and SFs that bind these regions, will be necessary to mechanistically elucidate how aging reshapes the brain transcriptome.

Our additional analyses aimed at dissecting the influence of different alternative splicing events indicate the presence of age-specific isoform switches. Notably, we observed in both our datasets a higher fraction of genes with alternative 3′ acceptor sites (A3) in the aged brain, whereas the young adult brain displayed a higher fraction of genes with alternative transcription start sites (ATTS), suggesting that A3-type splicing events might protect isoforms from degradation while ATSS-type splicing events might have the opposite effect. Overall, in the aged brain the overexpressed isoforms belonging to the same gene also tend to be less sensitive to NMD (Figure 5D), possibly indicating a positive selection of NMD-resistant transcripts in the aged brain.

Analysis of mRNA PT ratio provided further insight into the transcriptomic changes that occur in the aging brain, revealing a scenario compatible with a significant increase in mRNA lifetime in aged mice (Figure 6B). These findings are consistent with recent estimates of elongation speed obtained in a recent work from Debès and collaborators (28). Their work showed a reduction in the fraction of unspliced reads with aging, which the authors interpret as increased splicing efficiency associated with faster PolII elongation. While plausible, the authors themselves acknowledged that they ‘cannot exclude the possibility that this increase resulted from changes in RNA half-lives’. Consistently, our study observed a similar reduction in the fraction of unspliced reads with age, yet we propose that this decrease results from the combined modulation of RNAs processing and stability. Our study expands on the insights provided by Debès and coworkers. regarding splicing modulation and introduces novel relevant evidence supporting the involvement of RNA decay in aging-related gene expression modulations, suggesting that mRNA turnover plays a role in defining transcriptional programs associated with brain aging. Given the alterations in alternative splicing that we have observed here and that seem to correlate with slower mRNA turnover in the aged brain, we believe that decreasing elongation speed may not have therapeutic potential in this context, but further experiments will be needed to test this aspect. As both mRNA (here) and protein lifetimes (31,127) have been found to be affected in the aged mouse brain, these findings highlight the importance of measures that not only reflect abundances but also the dynamic replacement of biomolecules in the aged brain to understand differences that might otherwise remain unexplored.

Among the limitations of our study, there is the fact that we concentrated our analysis on male individuals, limiting the impact of our work for sex-related differences. Historically it was believed that results in female mice, could be less reproducible, but this is probably not the case (128), and future studies will be required to bridge the gender gap in aging research. Our additional analysis in this direction tried to account for possible sex-chromosome related effects. In our male cohorts we did not observe a significant difference in the expression of X-chromosome or Y-chromosome specific genes. Of course, this does not rule out the possibility that there may be sex-specific differences in gene expression in the aged brain of male and female rodents, as previously shown (7), although additional experiments will be needed to address DTU and a possible role of RBPs in determining these differences. One other limitation of our work is that we lack a direct comparison with single-cell and spatial transcriptomics data within the same animal cohort, although we did a sizeable effort to re-use and integrate in our analyses a large wealth of recently generated datasets (6,37,39). Admittedly the single-cell data comparisons that we show lend support to our interpretations, but they do not completely rule out potential alterations in cell population composition. Finally, although our results indicate a clear implication of RBPs and SFs in the remodeling of the aged transcriptome, our binding motif analysis was performed *in silico* and future more extensive experimental efforts will be needed to directly test the role of these proteins in brain aging.

In conclusion, our work presents a systematic analysis of the aging mouse transcriptome at the level of the whole brain, revealing significant alterations in gene expression, isoform usage and mRNA turnover. By thorough analyses, we have identified age-specific changes in neuronal gene regulation, alternative splicing, and mRNA stability which opens up new avenues for future research into the molecular basis of brain aging and age-associated neurodegenerative diseases. These findings have important implications for the study of age-related neurodegenerative disorders, as they may uncover potential therapeutic targets and strategies for delaying or preventing age-associated cognitive decline, restoring the alteration of deregulated pathways.

## Supplementary Material

gkae172_Supplemental_Files

## Data Availability

The datasets generated and analyzed during in current study are available on GEO under the accession number: GSE233837 Note that the ‘super series’ GSE233837 contains three subseries: GSE233835: Short-read 150bp paired-end (main dataset for our analyses) GSE233836: Long-read Oxford Nanopore Technology (ONT) dataset GSE249499: Short-read 50bp single-end (second independent biological cohort used for cross-validations) The scripts utilized in the current study are also publicly available in Figshare at https://doi.org/10.6084/m9.figshare.25288339.v1.

## Reproducibility

All methods and analyses used in this study are thoroughly described to ensure the reproducibility and the datasets are deposited (see GSE233837, data availability section, FigShare and details about retrieving other available datasets described in the methods).

## References

[1] López-Otín C. , Blasco M.A., Partridge L., Serrano M., Kroemer G. Hallmarks of aging: an expanding universe. Cell. 2023; 186:243–278. 36599349 10.1016/j.cell.2022.11.001

[2] Mattson M.P. , Arumugam T.V. Hallmarks of brain aging: adaptive and pathological modification by metabolic states. Cell Metab. 2018; 27:1176–1199. 29874566 10.1016/j.cmet.2018.05.011 PMC6039826

[3] Peters A. The effects of normal aging on myelin and nerve fibers: a review. J. Neurocytol. 2002; 31:581–593. 14501200 10.1023/a:1025731309829

[4] Ham S. , Lee S.J.V. Advances in transcriptome analysis of human brain aging. Exp. Mol. Med. 2020; 52:1787–1797. 33244150 10.1038/s12276-020-00522-6 PMC8080664

[5] Wyss-Coray T. Ageing, neurodegeneration and brain rejuvenation. Nature. 2016; 539:180. 27830812 10.1038/nature20411 PMC5172605

[6] Almanzar N. , Antony J., Baghel A.S., Bakerman I., Bansal I., Barres B.A., Beachy P.A., Berdnik D., Bilen B., Brownfield D. et al. A single-cell transcriptomic atlas characterizes ageing tissues in the mouse. Nature. 2020; 583:590–595. 32669714 10.1038/s41586-020-2496-1 PMC8240505

[7] Trabzuni D. , Ramasamy A., Imran S., Walker R., Smith C., Weale M.E., Hardy J., Ryten M. Widespread sex differences in gene expression and splicing in the adult human brain. Nat. Commun. 2013; 4:2771. 24264146 10.1038/ncomms3771 PMC3868224

[8] Ori A. , Toyama B.H., Harris M.S., Bock T., Iskar M., Bork P., Ingolia N.T., Hetzer M.W., Beck M. Integrated transcriptome and proteome analyses reveal organ-specific proteome deterioration in old rats. Cell Syst. 2015; 1:224–237. 27135913 10.1016/j.cels.2015.08.012 PMC4802414

[9] Izgi H. , Han D., Isildak U., Huang S., Kocabiyik E., Khaitovich P., Somel M., Dönertaş H.M. Inter-tissue convergence of gene expression during ageing suggests age-related loss of tissue and cellular identity. eLife. 2022; 11:e68048. 35098922 10.7554/eLife.68048 PMC8880995

[10] Soreq L. UK Brain Expression Consortium, North American Brain Expression Consortium UK Brain Expression Consortium, North American Brain Expression Consortium Rose J. , Soreq E., Hardy J., Trabzuni D., Cookson M.R., Smith C., Ryten M. et al. Major shifts in glial regional identity are a transcriptional hallmark of Human brain aging. Cell Rep. 2017; 18:557–570. 28076797 10.1016/j.celrep.2016.12.011 PMC5263238

[11] Cheng H. , Xuan H., Green C.D., Han Y., Sun N., Shen H., McDermott J., Bennett D.A., Lan F., Han J.D.J. Repression of human and mouse brain inflammaging transcriptome by broad gene-body histone hyperacetylation. Proc. Natl. Acad. Sci. U.S.A. 2018; 115:7611–7616. 29967166 10.1073/pnas.1800656115 PMC6055154

[12] Lu T. , Aron L., Zullo J., Pan Y., Kim H., Chen Y., Yang T.H., Kim H.M., Drake D., Liu X.S. et al. REST and stress resistance in ageing and Alzheimer's disease. Nature. 2014; 507:448–454. 24670762 10.1038/nature13163 PMC4110979

[13] Boisvert M.M. , Erikson G.A., Shokhirev M.N., Allen N.J. The aging astrocyte transcriptome from multiple regions of the mouse brain. Cell Rep. 2018; 22:269–285. 29298427 10.1016/j.celrep.2017.12.039 PMC5783200

[14] Shukla R. , Prevot T.D., French L., Isserlin R., Rocco B.R., Banasr M., Bader G.D., Sibille E. The relative contributions of cell-dependent cortical microcircuit aging to cognition and anxiety. Biol. Psychiatry. 2019; 85:257–267. 30446205 10.1016/j.biopsych.2018.09.019

[15] Srivastava A. , Barth E., Ermolaeva M.A., Guenther M., Frahm C., Marz M., Witte O.W. Tissue-specific gene expression changes are associated with aging in mice. Genomics Proteomics Bioinformatics. 2020; 18:430–442. 33309863 10.1016/j.gpb.2020.12.001 PMC8242333

[16] Sanchez D. , Bajo-Grañeras R., Del Caño-Espinel M., Garcia-Centeno R., Garcia-Mateo N., Pascua-Maestro R., Ganfornina M.D. Aging without apolipoprotein d: molecular and cellular modifications in the hippocampus and cortex. Exp. Gerontol. 2015; 67:19–47. 25868396 10.1016/j.exger.2015.04.003

[17] Stilling R.M. , Benito E., Gertig M., Barth J., Capece V., Burkhardt S., Bonn S., Fischer A. De-regulation of gene expression and alternative splicing affects distinct cellular pathways in the aging hippocampus. Front. Cell Neurosci. 2014; 8:373. 25431548 10.3389/fncel.2014.00373 PMC4230043

[18] Hu Y. , Pan J., Xin Y., Mi X., Wang J., Gao Q., Luo H. Gene expression analysis reveals novel gene signatures between young and old adults in human prefrontal cortex. Front. Aging Neurosci. 2018; 10:259. 30210331 10.3389/fnagi.2018.00259 PMC6119720

[19] Berchtold N.C. , Cribbs D.H., Coleman P.D., Rogers J., Head E., Kim R., Beach T., Miller C., Troncoso J., Trojanowski J.Q. et al. Gene expression changes in the course of normal brain aging are sexually dimorphic. Proc. Natl. Acad. Sci. U.S.A. 2008; 105:15605–15610. 18832152 10.1073/pnas.0806883105 PMC2563070

[20] Bae S.H. , Kim H.W., Shin S.J., Kim J., Jeong Y.H., Moon J. Decipher reliable biomarkers of brain aging by integrating literature-based evidence with interactome data. Exp. Mol. Med. 2018; 50:1–15. 10.1038/s12276-018-0057-6 PMC5938059 29651153

[21] Pan J. , Ma N., Yu B., Zhang W., Wan J. Transcriptomic profiling of microglia and astrocytes throughout aging. J. Neuroinflamm. 2020; 17:97. 10.1186/s12974-020-01774-9 PMC7115095 32238175

[22] Lee C.K. , Weindruch R., Prolla T.A. Gene-expression profile of the ageing brain in mice. Nat. Genet. 2000; 25:294–297. 10888876 10.1038/77046

[23] Wehrspaun C.C. , Haerty W., Ponting C.P. Microglia recapitulate a hematopoietic master regulator network in the aging human frontal cortex. Neurobiol. Aging. 2015; 36:2443.e9–2443.e20. 10.1016/j.neurobiolaging.2015.04.008 PMC4503803 26002684

[24] Pozniak C.D. , Barnabé-Heider F., Rymar V.V., Lee A.F., Sadikot A.F., Miller F.D. p73 is required for survival and maintenance of CNS neurons. J. Neurosci. 2002; 22:9800–9809. 12427836 10.1523/JNEUROSCI.22-22-09800.2002 PMC6757829

[25] Mangalmurti A. , Lukens J.R. How neurons die in Alzheimer's disease: implications for neuroinflammation. Curr. Opin. Neurobiol. 2022; 75:102575. 35691251 10.1016/j.conb.2022.102575 PMC9380082

[26] Cheung P. , Vallania F., Warsinske H.C., Donato M., Schaffert S., Chang S.E., Dvorak M., Dekker C.L., Davis M.M., Utz P.J. et al. Single-cell chromatin modification profiling reveals increased epigenetic variations with aging. Cell. 2018; 173:1385–1397. 29706550 10.1016/j.cell.2018.03.079 PMC5984186

[27] Pollina E.A. , Gilliam D.T., Landau A.T., Lin C., Pajarillo N., Davis C.P., Harmin D.A., Yap E.-L., Vogel I.R., Griffith E.C. et al. A NPAS4-NuA4 complex couples synaptic activity to DNA repair. Nature. 2023; 614:732–741. 36792830 10.1038/s41586-023-05711-7 PMC9946837

[28] Debès C. , Papadakis A., Grönke S., Karalay Ö., Tain L.S., Mizi A., Nakamura S., Hahn O., Weigelt C., Josipovic N. et al. Ageing-associated changes in transcriptional elongation influence longevity. Nature. 2023; 616:814–821. 37046086 10.1038/s41586-023-05922-y PMC10132977

[29] Walther D.M. , Mann M. Accurate quantification of more than 4000 mouse tissue proteins reveals minimal proteome changes during aging. Mol. Cell. Proteomics. 2011; 10:M110.004523. 10.1074/mcp.M110.004523 PMC3033683 21048193

[30] Kelmer Sacramento E. , Kirkpatrick J.M., Mazzetto M., Baumgart M., Bartolome A., Di Sanzo S., Caterino C., Sanguanini M., Papaevgeniou N., Lefaki M. et al. Reduced proteasome activity in the aging brain results in ribosome stoichiometry loss and aggregation. Mol. Syst. Biol. 2020; 16:e9596. 32558274 10.15252/msb.20209596 PMC7301280

[31] Kluever V. , Russo B., Mandad S., Kumar N.H., Alevra M., Ori A., Rizzoli S.O., Urlaub H., Schneider A., Fornasiero E.F. Protein lifetimes in aged brains reveal a proteostatic adaptation linking physiological aging to neurodegeneration. Sci. Adv. 2022; 8:eabn4437. 35594347 10.1126/sciadv.abn4437 PMC9122331

[32] Aebersold R. , Mann M. Mass spectrometry-based proteomics. Nature. 2003; 422:198–207. 12634793 10.1038/nature01511

[33] Smith L.M. , Kelleher N.L. Consortium for Top Down Proteomics Proteoform: a single term describing protein complexity. Nat. Methods. 2013; 10:186–187. 23443629 10.1038/nmeth.2369 PMC4114032

[34] Macek B. , Waanders L.F., Olsen J.V., Mann M. Top-down protein sequencing and MS3 on a hybrid linear quadrupole ion trap-orbitrap mass spectrometer. Mol. Cell. Proteomics. 2006; 5:949–958. 16478717 10.1074/mcp.T500042-MCP200

[35] Cupp-Sutton K.A. , Wu S. High-throughput quantitative top-down proteomics. Mol Omics. 2020; 16:91–99. 31932818 10.1039/c9mo00154a PMC7529119

[36] Yates J.R. , Kelleher N.L. Top down proteomics. Anal. Chem. 2013; 85:6151. 23705843 10.1021/ac401484r PMC4418644

[37] Allen W.E. , Blosser T.R., Sullivan Z.A., Dulac C., Zhuang X. Molecular and spatial signatures of mouse brain aging at single-cell resolution. Cell. 2023; 186:194–208. 36580914 10.1016/j.cell.2022.12.010 PMC10024607

[38] Armand E.J. , Li J., Xie F., Luo C., Mukamel E.A. Single-cell sequencing of brain cell transcriptomes and epigenomes. Neuron. 2021; 109:11–26. 33412093 10.1016/j.neuron.2020.12.010 PMC7808568

[39] Buckley M.T. , Sun E.D., George B.M., Liu L., Schaum N., Xu L., Reyes J.M., Goodell M.A., Weissman I.L., Wyss-Coray T. et al. Cell-type-specific aging clocks to quantify aging and rejuvenation in neurogenic regions of the brain. Nat. Aging. 2023; 3:121–137. 37118510 10.1038/s43587-022-00335-4 PMC10154228

[40] Arzalluz-Luqueángeles Á. , Conesa A Single-cell RNAseq for the study of isoforms-how is that possible?. Genome Biol. 2018; 19:110. 30097058 10.1186/s13059-018-1496-z PMC6085759

[41] Brown K.A. , Melby J.A., Roberts D.S., Ge Y. Top-down proteomics: challenges, innovations, and applications in basic and clinical research. Expert Rev. Proteomics. 2020; 17:719–733. 33232185 10.1080/14789450.2020.1855982 PMC7864889

[42] Kornblihtt A.R. , Schor I.E., Alló M., Dujardin G., Petrillo E., Muñoz M.J. Alternative splicing: a pivotal step between eukaryotic transcription and translation. Nat. Rev. Mol. Cell Biol. 2013; 14:153–165. 23385723 10.1038/nrm3525

[43] Melé M. , Ferreira P.G., Reverter F., DeLuca D.S., Monlong J., Sammeth M., Young T.R., Goldmann J.M., Pervouchine D.D., Sullivan T.J. et al. The human transcriptome across tissues and individuals. Science. 2015; 348:660–665. 25954002 10.1126/science.aaa0355 PMC4547472

[44] Su C.H. , Dhananjaya D., Tarn W.Y. Alternative splicing in neurogenesis and brain development. Front Mol. Biosci. 2018; 5:12. 29484299 10.3389/fmolb.2018.00012 PMC5816070

[45] Vuong C.K. , Black D.L., Zheng S. The neurogenetics of alternative splicing. Nat. Rev. Neurosci. 2016; 17:265–281. 27094079 10.1038/nrn.2016.27 PMC4861142

[46] Tushev G. , Glock C., Heumüller M., Biever A., Jovanovic M., Schuman E.M. Alternative 3′ UTRs modify the localization, regulatory potential, stability, and plasticity of mRNAs in neuronal compartments. Neuron. 2018; 98:495–511. 29656876 10.1016/j.neuron.2018.03.030

[47] Bae B. , Miura P. Emerging roles for 3′ UTRs in neurons. Int. J. Mol. Sci. 2020; 21:3413. 32408514 10.3390/ijms21103413 PMC7279237

[48] Wehrspaun C.C. , Ponting C.P., Marques A.C. Brain-expressed 3’UTR extensions strengthen miRNA cross-talk between ion channel/transporter encoding mRNAs. Front. Genet. 2014; 5:41. 24616735 10.3389/fgene.2014.00041 PMC3935148

[49] Reyes A. , Huber W. Alternative start and termination sites of transcription drive most transcript isoform differences across human tissues. Nucleic Acids Res. 2018; 46:582–592. 29202200 10.1093/nar/gkx1165 PMC5778607

[50] Rockenstein E.M. , McConlogue L., Tan H., Power M., Masliah E., Mucke L. Levels and alternative splicing of amyloid β protein precursor (APP) transcripts in brains of APP transgenic mice and humans with Alzheimer's disease. J. Biol. Chem. 1995; 270:28257–28267. 7499323 10.1074/jbc.270.47.28257

[51] Arai T. , Hasegawa M., Akiyama H., Ikeda K., Nonaka T., Mori H., Mann D., Tsuchiya K., Yoshida M., Hashizume Y. et al. TDP-43 is a component of ubiquitin-positive tau-negative inclusions in frontotemporal lobar degeneration and amyotrophic lateral sclerosis. Biochem. Biophys. Res. Commun. 2006; 351:602–611. 17084815 10.1016/j.bbrc.2006.10.093

[52] Dredge B.K. , Polydorides A.D., Darnell R.B. The splice of life: alternative splicing and neurological disease. Nat. Rev. Neurosci. 2001; 2:43–50. 11253358 10.1038/35049061

[53] Parikshak N.N. , Swarup V., Belgard T.G., Irimia M., Ramaswami G., Gandal M.J., Hartl C., Leppa V., Ubieta L.D.L.T., Huang J. et al. Genome-wide changes in lncRNA, splicing, and regional gene expression patterns in autism. Nature. 2016; 540:423–427. 27919067 10.1038/nature20612 PMC7102905

[54] Mazin P. , Xiong J., Liu X., Yan Z., Zhang X., Li M., He L., Somel M., Yuan Y., Phoebe Chen Y.P. et al. Widespread splicing changes in human brain development and aging. Mol. Syst. Biol. 2013; 9:633. 23340839 10.1038/msb.2012.67 PMC3564255

[55] Gooding C. , Roberts G.C., Smith C.W.J. Role of an inhibitory pyrimidine element and polypyrimidine tract binding protein in repression of a regulated alpha-tropomyosin exon. RNA. 1998; 4:85. 9436911 PMC1369599

[56] Tollervey J.R. , Wang Z., Hortobágyi T., Witten J.T., Zarnack K., Kayikci M., Clark T.A., Schweitzer A.C., Rot G., Curk T. et al. Analysis of alternative splicing associated with aging and neurodegeneration in the human brain. Genome Res. 2011; 21:1572–1582. 21846794 10.1101/gr.122226.111 PMC3202275

[57] Raj T. , Li Y.I., Wong G., Humphrey J., Wang M., Ramdhani S., Wang Y.-C., Ng B., Gupta I., Haroutunian V. et al. Integrative transcriptome analyses of the aging brain implicate altered splicing in Alzheimer's disease susceptibility. Nat. Genet. 2018; 50:1584–1592. 30297968 10.1038/s41588-018-0238-1 PMC6354244

[58] Trabzuni D. , Wray S., Vandrovcova J., Ramasamy A., Walker R., Smith C., Luk C., Gibbs J.R., Dillman A., Hernandez D.G. et al. MAPT expression and splicing is differentially regulated by brain region: relation to genotype and implication for tauopathies. Hum. Mol. Genet. 2012; 21:4094–4103. 22723018 10.1093/hmg/dds238 PMC3428157

[59] Leung S.K. , Jeffries A.R., Castanho I., Jordan B.T., Moore K., Davies J.P., Dempster E.L., Bray N.J., O’Neill P., Tseng E. et al. Full-length transcript sequencing of human and mouse cerebral cortex identifies widespread isoform diversity and alternative splicing. Cell Rep. 2021; 37:110022. 34788620 10.1016/j.celrep.2021.110022 PMC8609283

[60] Yu Y. , Fuscoe J.C., Zhao C., Guo C., Jia M., Qing T., Bannon D.I., Lancashire L., Bao W., Du T. et al. A rat RNA-seq transcriptomic BodyMap across 11 organs and 4 developmental stages. Nat. Commun. 2014; 5:3230. 24510058 10.1038/ncomms4230 PMC3926002

[61] Marques-Coelho D. , Iohan L., da C.C., Melo de Farias A.R., Flaig A., Letournel F., Martin-Négrier M.L., Chapon F., Faisant M., Godfraind C. et al. Differential transcript usage unravels gene expression alterations in Alzheimer's disease human brains. NPJ Aging Mech. Dis. 2021; 7:2. 33398016 10.1038/s41514-020-00052-5 PMC7782705

[62] Street L. , Rothamel K., Brannan K., Jin W., Bokor B., Dong K., Rhine K., Madrigal A., Al-Azzam N., Kim J.K. et al. Large-scale map of RNA binding protein interactomes across the mRNA life-cycle. 2023; bioRxiv doi:08 June 2023, preprint: not peer reviewed 10.1101/2023.06.08.544225. PMC11530141 39303721

[63] Lorenz D.A. , Her H.-L., Shen K.A., Rothamel K., Hutt K.R., Nojadera A.C., Bruns S.C., Manakov S.A., Yee B.A., Chapman K.B. et al. Multiplexed transcriptome discovery of RNA-binding protein binding sites by antibody-barcode eCLIP. Nat. Methods. 2023; 20:65–69. 36550273 10.1038/s41592-022-01708-8 PMC9834051

[64] Fornasiero E.F. , Mandad S., Wildhagen H., Alevra M., Rammner B., Keihani S., Opazo F., Urban I., Ischebeck T., Sakib M.S. et al. Precisely measured protein lifetimes in the mouse brain reveal differences across tissues and subcellular fractions. Nat. Commun. 2018; 9:4230. 30315172 10.1038/s41467-018-06519-0 PMC6185916

[65] Coffey C.E. , Lucke J.F., Saxton J.A., Ratcliff G., Unitas L.J., Billig B., Bryan R.N. Sex differences in brain aging: a quantitative magnetic resonance imaging study. Arch. Neurol. 1998; 55:169–179. 9482358 10.1001/archneur.55.2.169

[66] Rahimi K. , Venø M.T., Dupont D.M., Kjems J. Nanopore sequencing of brain-derived full-length circRNAs reveals circRNA-specific exon usage, intron retention and microexons. Nat. Commun. 2021; 12:4825. 34376658 10.1038/s41467-021-24975-z PMC8355340

[67] Bentley D.R. , Balasubramanian S., Swerdlow H.P., Smith G.P., Milton J., Brown C.G., Hall K.P., Evers D.J., Barnes C.L., Bignell H.R. et al. Accurate whole human genome sequencing using reversible terminator chemistry. Nature. 2008; 456:53–59. 18987734 10.1038/nature07517 PMC2581791

[68] Andrews S. FASTQC. A quality control tool for high throughput sequence data. 2010; http://www.bioinformatics.babraham.ac.uk/projects/fastqc.

[69] Martin M. Cutadapt removes adapter sequences from high-throughput sequencing reads. EMBnet. J. 2011; 17:10–12.

[70] Kim D. , Langmead B., Salzberg S.L. HISAT: a fast spliced aligner with low memory requirements. Nat. Methods. 2015; 12:357–360. 25751142 10.1038/nmeth.3317 PMC4655817

[71] Liao Y. , Smyth G.K., Shi W. The subread aligner: fast, accurate and scalable read mapping by seed-and-vote. NucleicAcids Res. 2013; 41:e108. 10.1093/nar/gkt214 PMC3664803 23558742

[72] Patro R. , Duggal G., Love M.I., Irizarry R.A., Kingsford C. Salmon provides fast and bias-aware quantification of transcript expression. Nat. Methods. 2017; 14:417–419. 28263959 10.1038/nmeth.4197 PMC5600148

[73] De Coster W. , D’Hert S., Schultz D.T., Cruts M., Van Broeckhoven C NanoPack: visualizing and processing long-read sequencing data. Bioinformatics. 2018; 34:2666–2669. 29547981 10.1093/bioinformatics/bty149 PMC6061794

[74] Li H. Minimap2: pairwise alignment for nucleotide sequences. Bioinformatics. 2018; 34:3094–3100. 29750242 10.1093/bioinformatics/bty191 PMC6137996

[75] Love M.I. , Huber W., Anders S. Moderated estimation of fold change and dispersion for RNA-seq data with DESeq2. Genome Biol. 2014; 15:550. 25516281 10.1186/s13059-014-0550-8 PMC4302049

[76] Wu T. , Hu E., Xu S., Chen M., Guo P., Dai Z., Feng T., Zhou L., Tang W., Zhan L. et al. clusterProfiler 4.0: a universal enrichment tool for interpreting omics data. The Innovation. 2021; 2:100141. 34557778 10.1016/j.xinn.2021.100141 PMC8454663

[77] Langfelder P. , Horvath S. WGCNA: an R package for weighted correlation network analysis. BMC Bioinf. 2008; 9:559. 10.1186/1471-2105-9-559 PMC2631488 19114008

[78] Shen L. of Icahn School of Medicine at Mount Sinai GeneOverlap: test and visualize gene overlaps. 2020;

[79] Hernandez D.G. , Nalls M.A., Moore M., Chong S., Dillman A., Trabzuni D., Gibbs J.R., Ryten M., Arepalli S., Weale M.E. et al. Integration of GWAS SNPs and tissue specific expression profiling reveal discrete eQTLs for human traits in blood and brain. Neurobiol. Dis. 2012; 47:20–28. 22433082 10.1016/j.nbd.2012.03.020 PMC3358430

[80] Vitting-Seerup K. , Sandelin A., Berger B. IsoformSwitchAnalyzeR: analysis of changes in genome-wide patterns of alternaive splicing and its functional consequences. Bioinformatics. 2019; 35:4469–4471. 30989184 10.1093/bioinformatics/btz247

[81] Paz I. , Kosti I., Ares M., Cline M., Mandel-Gutfreund Y. RBPmap: a web server for mapping binding sites of RNA-binding proteins. Nucleic Acids. Res. 2014; 42:W361–W367. 24829458 10.1093/nar/gku406 PMC4086114

[82] Koopmans F. , van Nierop P., Andres-Alonso M., Byrnes A., Cijsouw T., Coba M.P., Cornelisse L.N., Farrell R.J., Goldschmidt H.L., Howrigan D.P. et al. SynGO: an evidence-based, expert-curated knowledge base for the synapse. Neuron. 2019; 103:217–234. 31171447 10.1016/j.neuron.2019.05.002 PMC6764089

[83] Ren Y. , Wang X., Zhang S., Hu H., Quicksall Z., Lee S., Morganti J.M., Johnson L.A., Asmann Y.W., Zhao N. Deconvolution reveals cell-type-specific transcriptomic changes in the aging mouse brain. Sci. Rep. 2023; 13:16855. 37803069 10.1038/s41598-023-44183-7 PMC10558435

[84] Thul P.J. , Lindskog C. The human protein atlas: a spatial map of the human proteome. Protein Sci. 2018; 27:233–244. 28940711 10.1002/pro.3307 PMC5734309

[85] LaForce G.R. , Farr J.S., Liu J., Akesson C., Gumus E., Pinkard O., Miranda H.C., Johnson K., Sweet T.J., Ji P. et al. Suppression of premature transcription termination leads to reduced mRNA isoform diversity and neurodegeneration. Neuron. 2022; 110:1340–1357. 35139363 10.1016/j.neuron.2022.01.018 PMC9035109

[86] Furlan M. , Galeota E., Gaudio N.D., Dassi E., Caselle M., de Pretis S., Pelizzola M Genome-wide dynamics of RNA synthesis, processing, and degradation without RNA metabolic labeling. Genome Res. 2020; 30:1492–1507. 32978246 10.1101/gr.260984.120 PMC7605262

[87] de Pretis S. , Kress T., Morelli M.J., Melloni G.E.M., Riva L., Amati B., Pelizzola M. INSPEcT: a computational tool to infer mRNA synthesis, processing and degradation dynamics from RNA- and 4sU-seq time course experiments. Bioinformatics. 2015; 31:2829–2835. 25957348 10.1093/bioinformatics/btv288

[88] Nichols N.R. , Day J.R., Laping N.J., Johnson S.A., Finch C.E. GFAP mRNA increases with age in rat and human brain. Neurobiol. Aging. 1993; 14:421–429. 8247224 10.1016/0197-4580(93)90100-p

[89] Porchet R. , Probst A., Bouras C., Dráberová E., Dráber P., Riederer B.M. Analysis of glial acidic fibrillary protein in the human entorhinal cortex during aging and in Alzheimer's disease. Proteomics. 2003; 3:1476–1485. 12923773 10.1002/pmic.200300456

[90] Wruck W. , Adjaye J. Meta-analysis of human prefrontal cortex reveals activation of GFAP and decline of synaptic transmission in the aging brain. Acta Neuropathol. Commun. 2020; 8:26. 32138778 10.1186/s40478-020-00907-8 PMC7059712

[91] Palmer A.L. , Ousman S.S. Astrocytes and aging. Front. Aging Neurosci. 2018; 10:419245. 10.3389/fnagi.2018.00337 PMC6212515 30416441

[92] Smirnov D. , Eremenko E., Stein D., Kaluski S., Jasinska W., Cosentino C., Martinez-Pastor B., Brotman Y., Mostoslavsky R., Khrameeva E. et al. SIRT6 is a key regulator of mitochondrial function in the brain. Cell Death. Dis. 2023; 14:35. 36653345 10.1038/s41419-022-05542-w PMC9849342

[93] Manczak M. , Jung Y., Park B.S., Partovi D., Reddy P.H. Time-course of mitochondrial gene expressions in mice brains: implications for mitochondrial dysfunction, oxidative damage, and cytochrome c in aging. J. Neurochem. 2005; 92:494–504. 15659220 10.1111/j.1471-4159.2004.02884.x

[94] Kastin A.J. , Akerstrom V. Entry of CART into brain is rapid but not inhibited by excess CART or leptin. Am. J. Physiol. 1999; 277:E901–E904. 10567018 10.1152/ajpendo.1999.277.5.E901

[95] Puelles E. , Rubenstein J.L., Puelles L. Chicken Nkx6.1 expression at advanced stages of development identifies distinct brain nuclei derived from the basal plate. Mech. Dev. 2001; 102:279–282. 11287211 10.1016/s0925-4773(01)00313-6

[96] Matsuura T. , Sutcliffe J.S., Fang P., Galjaard R.J., Jiang Y.H., Benton C.S., Rommens J.M., Beaudet A.L. De novo truncating mutations in E6-AP ubiquitin-protein ligase gene (UBE3A) in Angelman syndrome. Nat. Genet. 1997; 15:74–77. 8988172 10.1038/ng0197-74

[97] Kishino T. , Lalande M., Wagstaff J. UBE3A/E6-AP mutations cause Angelman syndrome. Nat. Genet. 1997; 15:70–73. 8988171 10.1038/ng0197-70

[98] Chidananda A.H. , Sharma A.K., Khandelwal R., Sharma Y. Secretagogin binding prevents α-synuclein fibrillation. Biochemistry. 2019; 58:4585–4589. 31617346 10.1021/acs.biochem.9b00656

[99] Flurkey K. , Currer J.M., Harrison D.E. Mouse models in aging research. Mouse Biomed. Res. 2007; 3:637–672.

[100] Cui J. , Shen N., Lu Z., Xu G., Wang Y., Jin B. Analysis and comprehensive comparison of PacBio and nanopore-based RNA sequencing of the Arabidopsis transcriptome. Plant Methods. 2020; 16:85. 32536962 10.1186/s13007-020-00629-x PMC7291481

[101] Engelhard C.A. , Khani S., Derdak S., Bilban M., Kornfeld J.-W. Nanopore sequencing unveils the complexity of the cold-activated murine brown adipose tissue transcriptome. iScience. 2023; 26:107190. 37564700 10.1016/j.isci.2023.107190 PMC10410515

[102] Südhof T.C. Towards an understanding of synapse formation. Neuron. 2018; 100:276–293. 30359597 10.1016/j.neuron.2018.09.040 PMC6226307

[103] Stoeger T. , Grant R.A., McQuattie-Pimentel A.C., Anekalla K.R., Liu S.S., Tejedor-Navarro H., Singer B.D., Abdala-Valencia H., Schwake M., Tetreault M.-P. et al. Aging is associated with a systemic length-associated transcriptome imbalance. Nature Aging. 2022; 2:1191–1206. 37118543 10.1038/s43587-022-00317-6 PMC10154227

[104] Kudla G. , Lipinski L., Caffin F., Helwak A., Zylicz M. High guanine and cytosine content increases mRNA levels in mammalian cells. PLoS Biol. 2006; 4:e180. 16700628 10.1371/journal.pbio.0040180 PMC1463026

[105] Hia F. , Yang S.F., Shichino Y., Yoshinaga M., Murakawa Y., Vandenbon A., Fukao A., Fujiwara T., Landthaler M., Natsume T. et al. Codon bias confers stability to human mRNAs. EMBO Rep. 2019; 20:e48220. 31482640 10.15252/embr.201948220 PMC6831995

[106] Sapir T. , Kshirsagar A., Gorelik A., Olender T., Porat Z., Scheffer I.E., Goldstein D.B., Devinsky O., Reiner O. Heterogeneous nuclear ribonucleoprotein U (HNRNPU) safeguards the developing mouse cortex. Nat. Commun. 2022; 13:4209. 35864088 10.1038/s41467-022-31752-z PMC9304408

[107] Fujinami H. , Shiraishi H., Hada K., Inoue M., Morisaki I., Higa R., Shin T., Kobayashi T., Hanada R., Penninger J.M. et al. CLP1 acts as the main RNA kinase in mice. Biochem. Biophys. Res. Commun. 2020; 10.1016/j.bbrc.2020.02.066. 32081435

[108] Kluever V. , Fornasiero E.F. Principles of brain aging: status and challenges of modeling human molecular changes in mice. Ageing Res. Rev. 2021; 72:101465. 34555542 10.1016/j.arr.2021.101465

[109] Irizar P.A. , Schäuble S., Esser D., Groth M., Frahm C., Priebe S., Baumgart M., Hartmann N., Marthandan S., Menzel U. et al. Transcriptomic alterations during ageing reflect the shift from cancer to degenerative diseases in the elderly. Nat. Commun. 2018; 9:327. 29382830 10.1038/s41467-017-02395-2 PMC5790807

[110] Hammelrath L. , Škokić S., Khmelinskii A., Hess A., van der Knaap N., Staring M., Lelieveldt B.P.F., Wiedermann D., Hoehn M. Morphological maturation of the mouse brain: an in vivo MRI and histology investigation. Neuroimage. 2016; 125:144–152. 26458518 10.1016/j.neuroimage.2015.10.009

[111] Fard M.K. , Van der Meer F., Sánchez P., Cantuti-Castelvetri L., Mandad S., Jäkel S., Fornasiero E.F., Schmitt S., Ehrlich M., Starost L. et al. BCAS1 expression defines a population of early myelinating oligodendrocytes in multiple sclerosis lesions. Sci. Transl. Med. 2017; 9:eaam7816. 29212715 10.1126/scitranslmed.aam7816 PMC7116798

[112] Lucin K.M. , Wyss-Coray T. Immune activation in brain aging and neurodegeneration: too much or too little?. Neuron. 2009; 64:110. 19840553 10.1016/j.neuron.2009.08.039 PMC2834890

[113] Antignano I. , Liu Y., Offermann N., Capasso M. Aging microglia. Cell. Mol. Life Sci. 2023; 80:126. 37081238 10.1007/s00018-023-04775-y PMC10119228

[114] Gyenis A. , Chang J., Demmers J.J.P.G., Bruens S.T., Barnhoorn S., Brandt R.M.C., Baar M.P., Raseta M., Derks K.W.J., Hoeijmakers J.H.J. et al. Genome-wide RNA polymerase stalling shapes the transcriptome during aging. Nat. Genet. 2023; 55:268–279. 36658433 10.1038/s41588-022-01279-6 PMC9925383

[115] von Bartheld C.S. Myths and truths about the cellular composition of the human brain: a review of influential concepts. J. Chem. Neuroanat. 2018; 93:2–15. 28873338 10.1016/j.jchemneu.2017.08.004 PMC5834348

[116] Peters A. , Morrison J.H., Rosene D.L., Hyman B.T. Feature article: are neurons lost from the primate cerebral cortex during normal aging?. Cereb. Cortex. 1998; 8:295–300. 9651126 10.1093/cercor/8.4.295

[117] West M.J. Regionally specific loss of neurons in the aging human hippocampus. Neurobiol. Aging. 1993; 14:287–293. 8367010 10.1016/0197-4580(93)90113-p

[118] Edler M.K. , Munger E.L., Meindl R.S., Hopkins W.D., Ely J.J., Erwin J.M., Mufson E.J., Hof P.R., Sherwood C.C., Raghanti M.A. Neuron loss associated with age but not Alzheimer's disease pathology in the chimpanzee brain. Philos. Trans. R. Soc. Lond. B Biol. Sci. 2020; 375:20190619. 32951541 10.1098/rstb.2019.0619 PMC7540958

[119] Boldrini M. , Fulmore C.A., Tartt A.N., Simeon L.R., Pavlova I., Poposka V., Rosoklija G.B., Stankov A., Arango V., Dwork A.J. et al. Human hippocampal neurogenesis persists throughout aging. Cell Stem Cell. 2018; 22:589–599. 29625071 10.1016/j.stem.2018.03.015 PMC5957089

[120] Han Y. , Kim S.Y. Endothelial senescence in vascular diseases: current understanding and future opportunities in senotherapeutics. Exp. Mol. Med. 2023; 55:1–12. 36599934 10.1038/s12276-022-00906-w PMC9898542

[121] Mohan K. , Gasparoni G., Salhab A., Orlich M.M., Geffers R., Hoffmann S., Adams R.H., Walter J., Nordheim A. Age-associated changes in endothelial transcriptome and epigenetic landscapes correlate with elevated risk of cerebral microbleeds. J. Am. Heart Assoc. 2023; 12:e031044. 37609982 10.1161/JAHA.123.031044 PMC10547332

[122] Ting K.K. , Coleman P., Zhao Y., Vadas M.A., Gamble J.R. The aging endothelium. Vasc Biol. 2021; 3:R35–R47. 33880430 10.1530/VB-20-0013 PMC8052565

[123] Ximerakis M. , Holton K.M., Giadone R.M., Ozek C., Saxena M., Santiago S., Adiconis X., Dionne D., Nguyen L., Shah K.M. et al. Heterochronic parabiosis reprograms the mouse brain transcriptome by shifting aging signatures in multiple cell types. Nat Aging. 2023; 3:327–345. 37118429 10.1038/s43587-023-00373-6 PMC10154248

[124] Wang W. , Zhao F., Ma X., Perry G., Zhu X. Mitochondria dysfunction in the pathogenesis of Alzheimer's disease: recent advances. Mol Neurodegener. 2020; 15:30. 32471464 10.1186/s13024-020-00376-6 PMC7257174

[125] Chen Z. , Zhong C. Oxidative stress in Alzheimer's disease. Neurosci Bull. 2014; 30:271–281. 24664866 10.1007/s12264-013-1423-y PMC5562667

[126] Ibañez-Solé O. , Barrio I., Izeta A. Age or lifestyle-induced accumulation of genotoxicity is associated with a length-dependent decrease in gene expression. iScience. 2023; 26:106368. 37013186 10.1016/j.isci.2023.106368 PMC10066539

[127] Rao N.R. , Upadhyay A., Savas J.N. Derailed protein turnover in the aging mammalian brain. Mol. Syst. Biol. 2024; 20:120–139. 38182797 10.1038/s44320-023-00009-2 PMC10897147

[128] Zajitschek S.R. , Zajitschek F., Bonduriansky R., Brooks R.C., Cornwell W., Falster D.S., Lagisz M., Mason J., Senior A.M., Noble D.W. et al. Sexual dimorphism in trait variability and its eco-evolutionary and statistical implications. eLife. 2020; 9:e63170. 33198888 10.7554/eLife.63170 PMC7704105

