# Supplementary material for: Comprehensive transcriptome analysis reveals altered mRNA splicing and post-transcriptional changes in the aged mouse brain
Source: Nucleic Acids Res. 2024 Mar 12;52(6):2865–85. doi: 10.1093/nar/gkae172 (PMC11014377; doi:10.1093/nar/gkae172)
Supplement: gkae172_Supplemental_Files [file gkae172_supplemental_files.zip › Supplementary_file_Hemandhar_Kumar_et_al_no_changes[2].pdf]

Supplementary Tables

Supplementary Table 1: Short-read and long-read dataset statistics
Supplementary Table 2: Counts and Differential expression analysis (DEA) at the whole gene level for both short and long read datasets (related to Fig. 2 and Supplementary Fig. 1)
Supplementary Table 3: Comparison to published datasets
Supplementary Table 4: Comparison of bulk (this study), single-cell datasets and region-specific datasets (related to Supplementary Fig. 2, Supplementary Fig. 3 Supplementary Fig.4, 5)
Supplementary Table 5: WGNCA analysis and module preservation analysis (related to Fig. 3 & Supplementary Fig. 1e-g)
Supplementary Table 6: GO-ORA of the modules (related to Fig. 3d-g)
Supplementary Table 7: Association of modules to single-cell dataset (The Tabula Muris Consortium, 2020; related to Fig. 3h)
Supplementary Table 8: Gene Overlap analysis for the human pathology (related to Fig. 3i)
Supplementary Table 9: Differential transcript expression (related to Fig. 4, Supplementary Fig. 11)
Supplementary Table 10: List of genes that have isoforms that are bidirectionally expressed (related to Fig. 4, Supplementary Fig. 11)
Supplementary Table 11: Differential transcript usage analysis (related to Fig. 5, Supplementary Fig. 11b)
Supplementary Table 12: RNA binding protein analysis (related to Supplementary Fig. 11e, 12)
Supplementary Table 13: RNA dynamics (related to Fig. 6)

Supplementary Figures
Supplementary Fig. 1: Summary of long-read data, module preservation and identified GOs
Supplementary Fig. 2: Comparison of mRNA expression between bulk and previously published single-cell dataset from the *Tabula muris senis* database
Supplementary Fig. 3: Detailed analysis of cell-type specific age-related changes in single-cell RNAseq datasets from mouse brain and comparison of microglial results to out short-read dataset
Supplementary Fig. 4: Comparison of bulk (this study, short-read) and region-specific mice dataset
Supplementary Fig. 5: Comparison of bulk (this study, short-read) and region-specific human dataset
Supplementary Fig. 6: String network for the M1 module
Supplementary Fig. 7: String network for the M2 module
Supplementary Fig. 8: String network for the M3 module
Supplementary Fig. 9: String network for the M8 module
Supplementary Fig. 10: Cross-validation of the results in a new animal cohort
Supplementary Fig. 11: Alternative splicing events and binding probability analysis of RNA-binding proteins (RBPs) and splicing factors (SFs) for cDNA
Supplementary Fig. 12: Binding probability analysis of RNA-binding proteins (RBPs) and splicing factors (SFs) for 3'UTR
Supplementary Fig. 13: RNA dynamics comparison between 6m and 24m mice

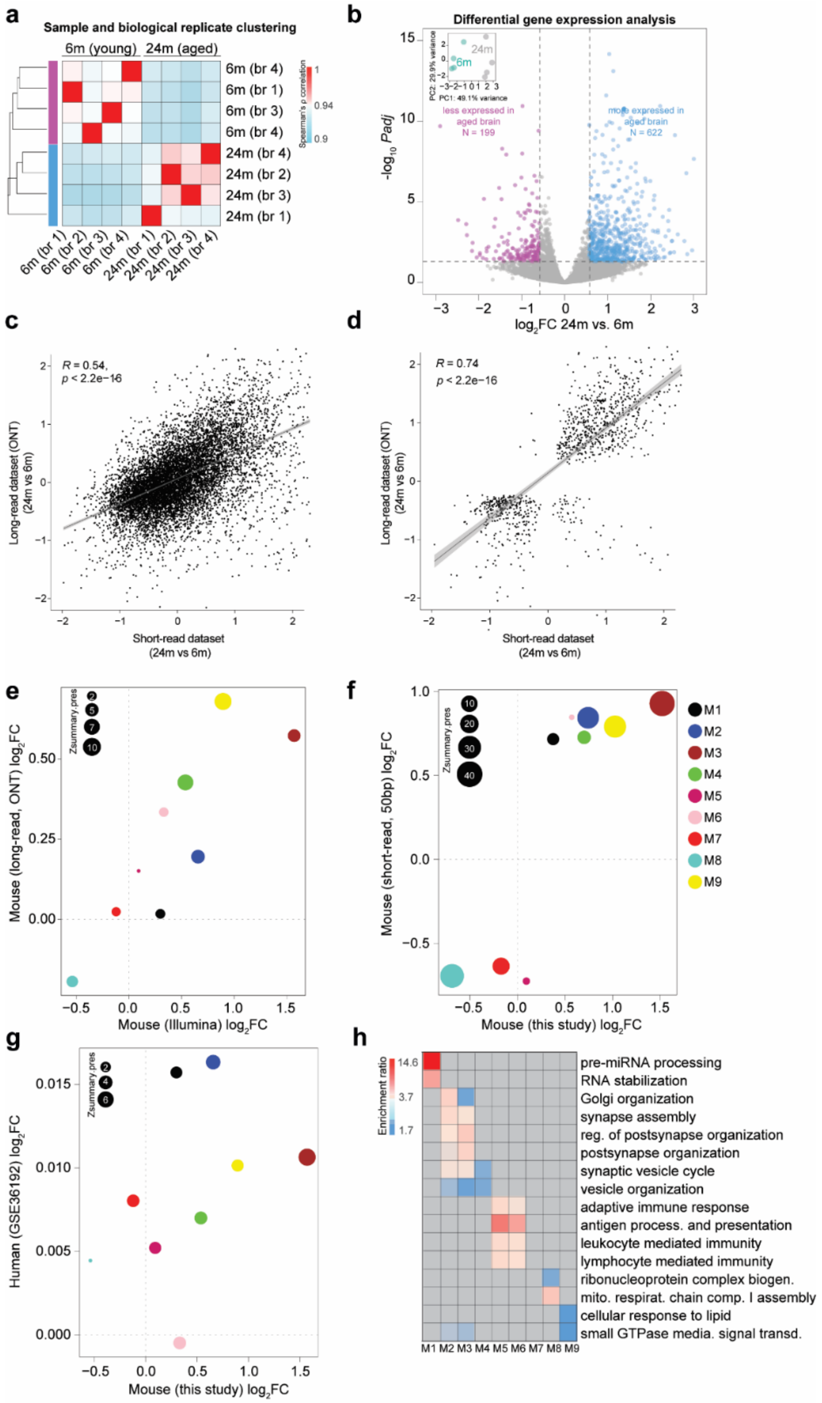

Supplementary Fig. 1: Summary of long-read data, module preservation and identified GOs. (**a**) Correlation matrix (spearman ρ correlation) of the long-read ONT dataset. (**b**) Volcano plot displaying DEGs in the long-read dataset, and a dot plot for the variance calculated using PCA. (c, d) Spearman ρ coefficients summarizing the correlations of log$_2$fold change differences for (c) all genes and (d) genes significant in both short-read dataset and long-read dataset (ONT) comparing 24m vs 6m. Points represent the log$_2$fold changes (Spearman's p-values). (**e-g**) Module preservation of Illumina database in (**e**) long-read ONT (**f**) a second independent animal cohort (**g**) human brain aging dataset (GSE36192 (7, 79)), each module is represented by a color code. The size of the bubble represents the Z-summary preservation statistic, Z-summary <2 indicate no evidence for module preservation, 2<Z-summary<10 indicates moderate evidence and Z-summary >10 indicates strong evidence for module preservation. The aging modules M1, M2, M3, M4, and M9 are preserved in both ONT and human datasets and also have a similar expression. (**h**) GO-ORA of all modules detected using the WGNCA method. Gray indicates not significant GOs.

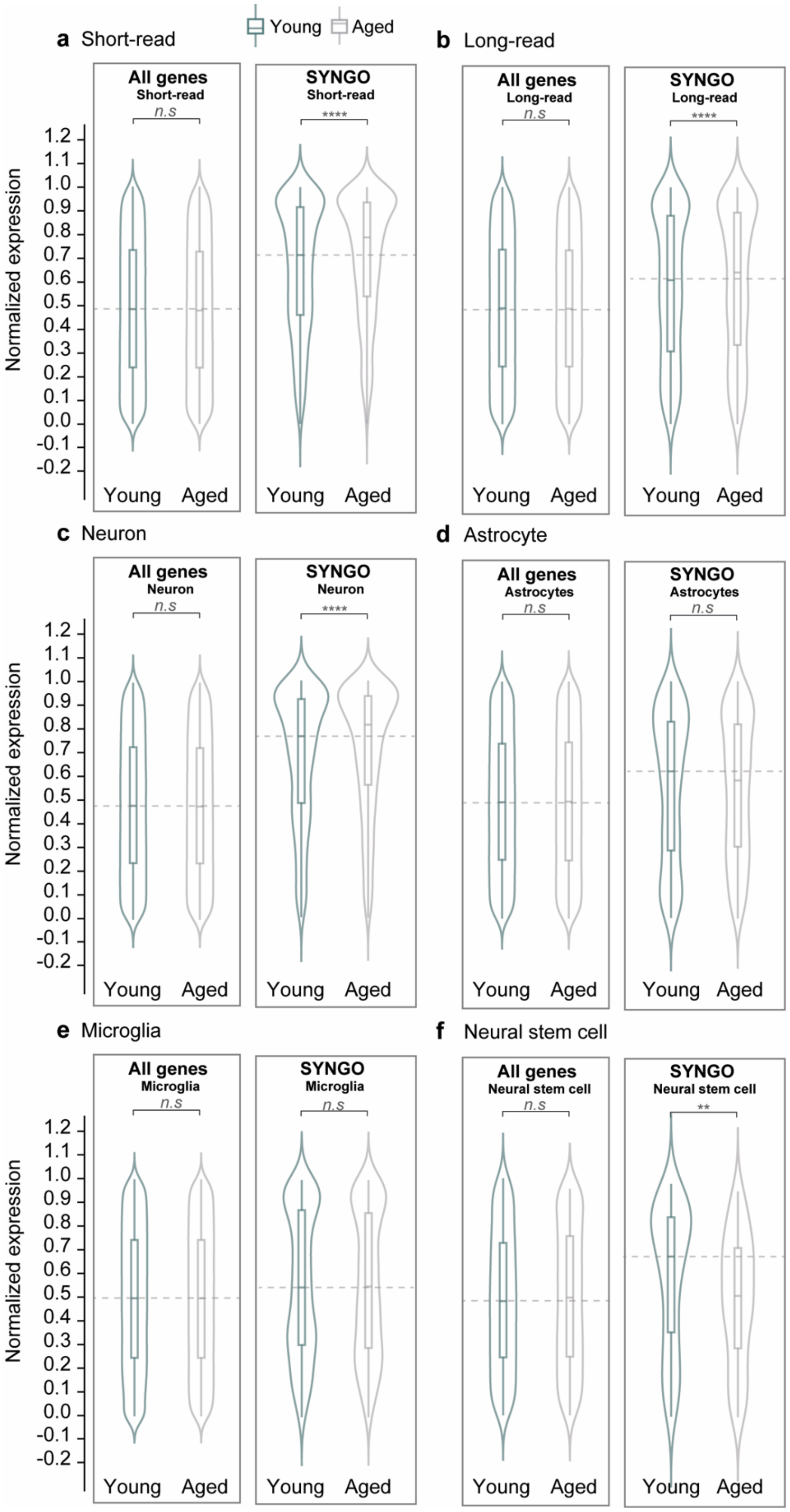

Supplementary Fig. 2: Comparison of mRNA expression between bulk and previously published single-cell dataset from the *Tabula muris senis* database (6). Average expression of gene curated in SynGO (82) database in (**a**) our short-read dataset (young 6m, aged 24m) and (**b-f**) Single-cell dataset, the **(c)** neuron, **(d)** astrocyte, **(e)** microglia, and **(f)** neuronal stem cell (young 3m, aged(6) 24m) (paired t-test, * ≤ 0.05, ** ≤ 0.01, *** ≤ 0.001, and **** ≤ 0.0001).

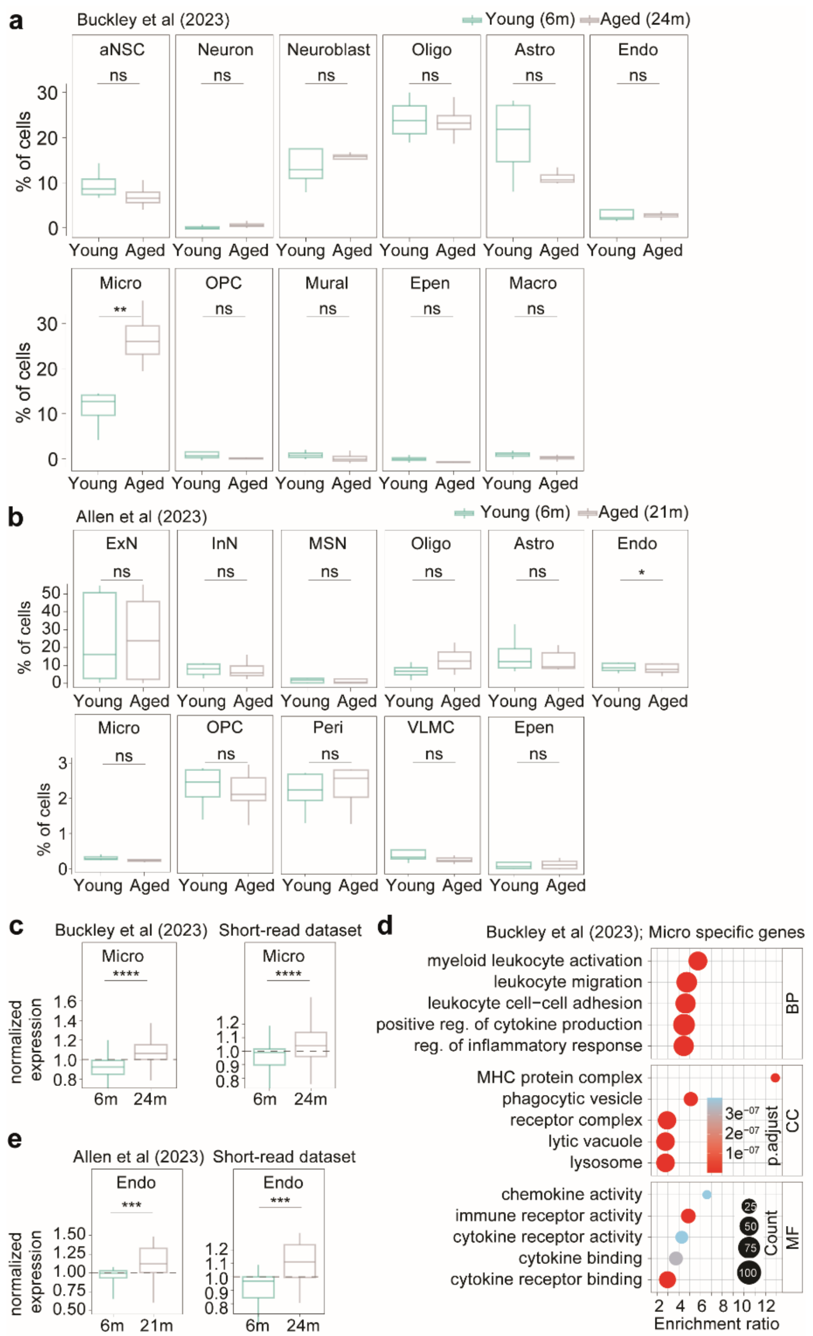

Supplementary Fig. 3: **Detailed analysis of cell-type specific age-related changes in single-cell RNAseq datasets from mouse brain and comparison of microglial results to out short-read dataset.** (**a,b**) Changes in cell number during aging in previously-published single-cell datasets, subdivided by cell type (37, 39). Abbreviations: activated neuronal stem cells (aNSC), oligodendrocytes (Oligo), astrocytes (Astro), endothelial cells (Endo), microglia (Micro), oligodendrocyte precursor cells (OPC), ependymal cells (Epen), macrophages (Macro), excitatory neurons (ExN), inhibitory neurons (InN), medium spiny neurons (MSN), pericytes (Peri), vascular leptomeningeal cells (VLMC). (**c**) Comparison of normalized expression for microglia specific genes across ages. **(d)** Gene ontology over-representation analysis (GO-ORA) for the microglia specific genes, shown here are top 5 GO terms based on the enrichment ratio. For all GO-ORA results in detail refer to Supplementary Table 4. Circle sizes in the enrichment graphs correspond to the number of terms for each GO term, and color scales represent the *padj.* (**e**) Comparison of normalized expression for endothelial specific genes across ages. For all comparisons, paired t-test, * ≤ 0.05, ** ≤ 0.01, *** ≤ 0.001, and **** ≤ 0.0001.

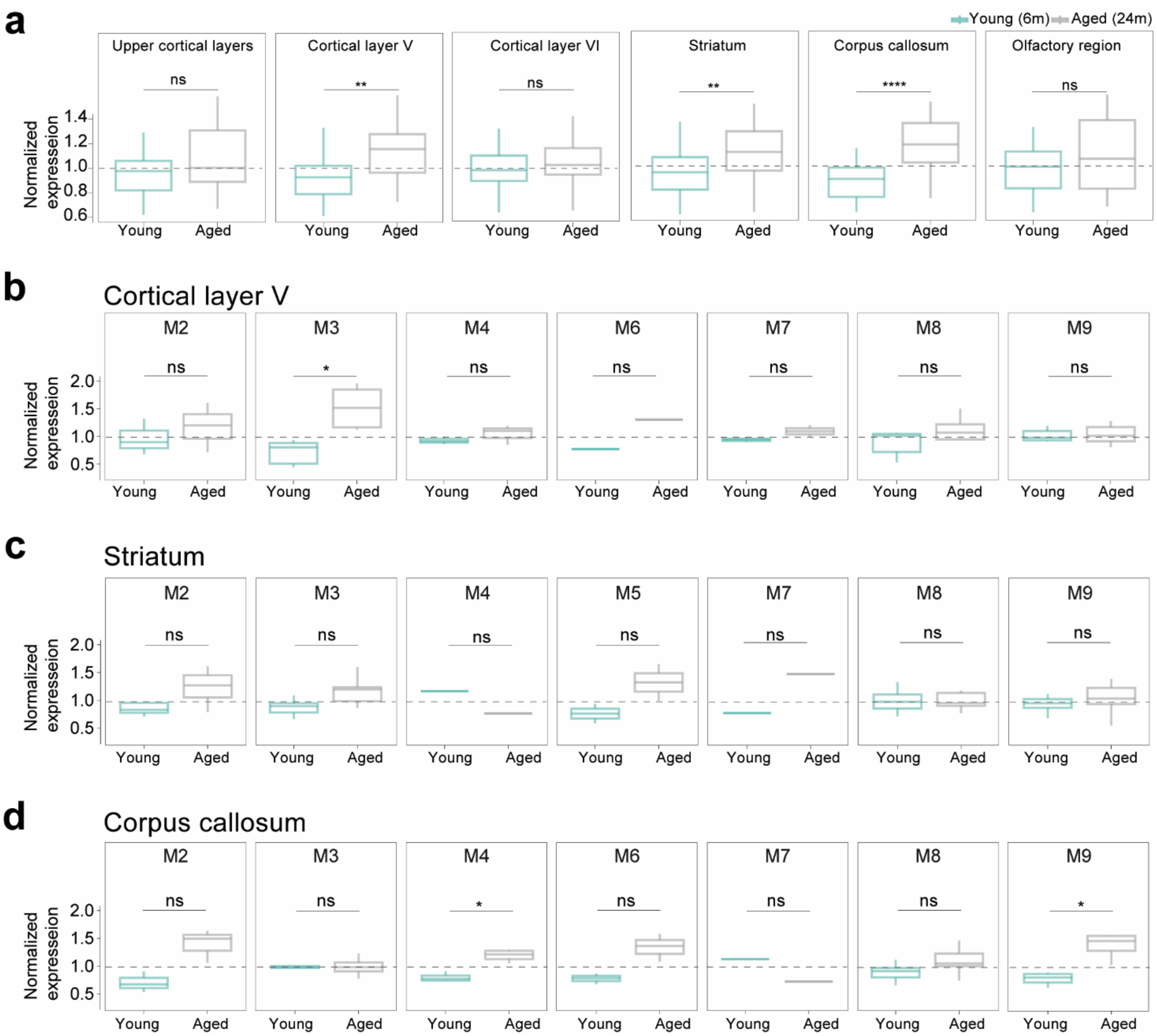

Supplementary Fig. 4: **Comparison of bulk (this study, short-read) and region-specific mice dataset. (a)** Normalized expression of region-specific genes defined from Allen and colleagues (37) in our short-read dataset. **(b-d)** Comparison of region-specific genes across modules (**b**) Cortical layer V, (**c**) Striatum and (**d**) Corpus callosum (paired t-test: * ≤ 0.05, ** ≤ 0.01, *** ≤ 0.001, and **** ≤ 0.0001).

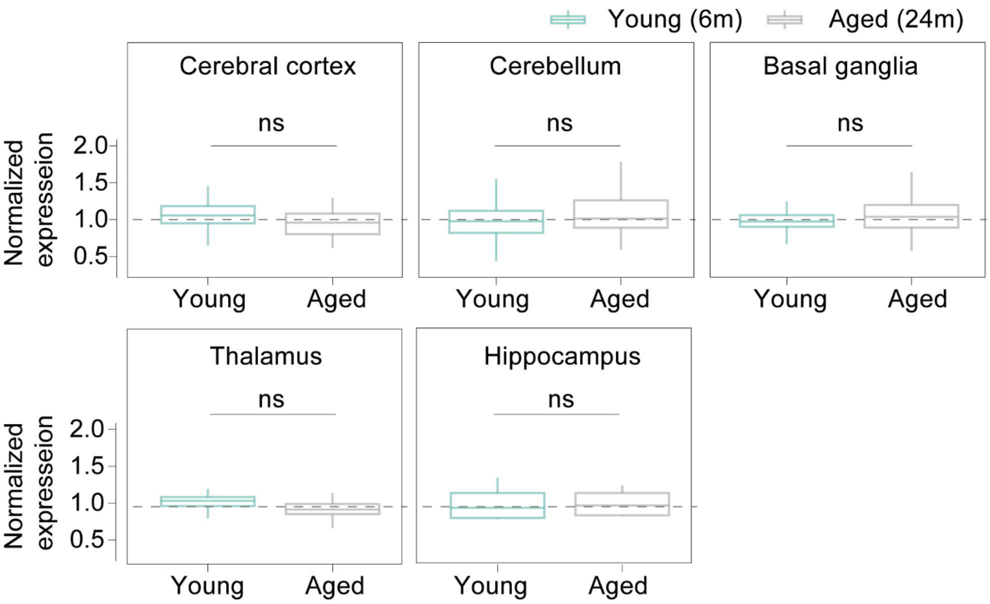

Supplementary Fig. 5: Comparison of bulk (this study, short-read) and region-specific human dataset. Normalized expression of region-specific genes from Human protein atlas (84) in short-read dataset (paired t-test: * ≤ 0.05, ** ≤ 0.01, *** ≤ 0.001, and **** ≤ 0.0001).

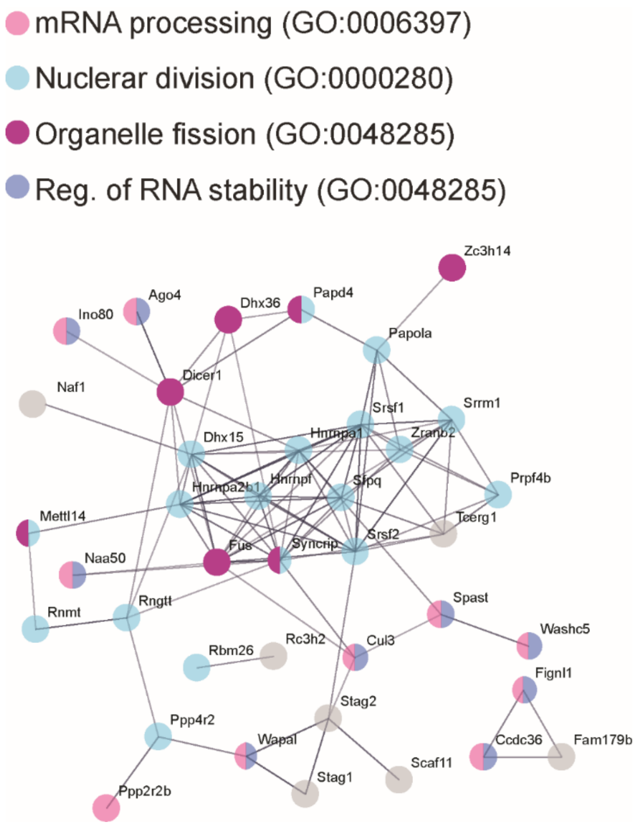

Supplementary Fig. 6: String network for the M1 module (inset in Fig. 3d). The legends in the upper part of the panel clarify their association with the respective pathways.

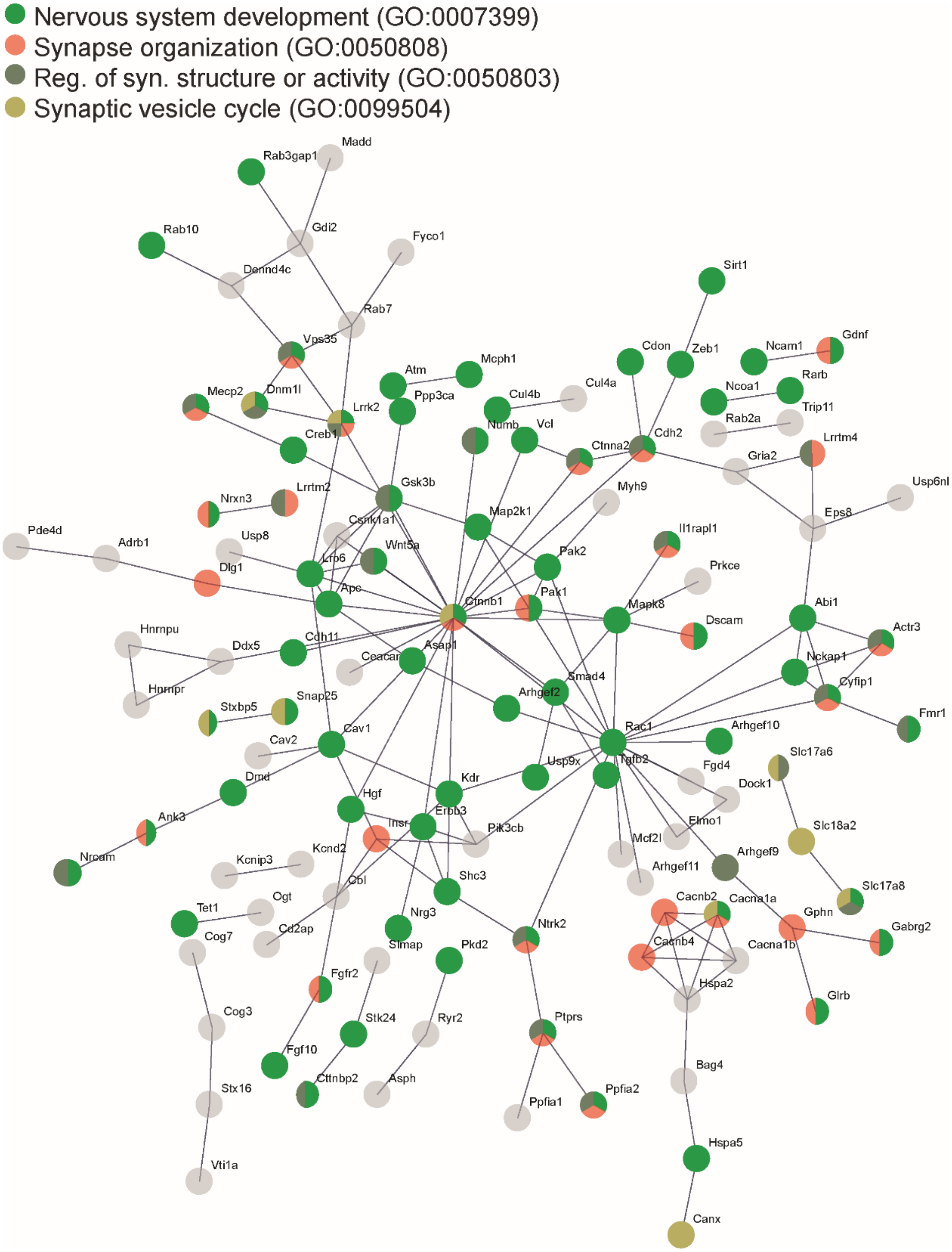

Supplementary Fig. 7: String network for the M2 module (inset in Fig. 3e). The legends in the upper part of the panel clarify their association with the respective pathways.

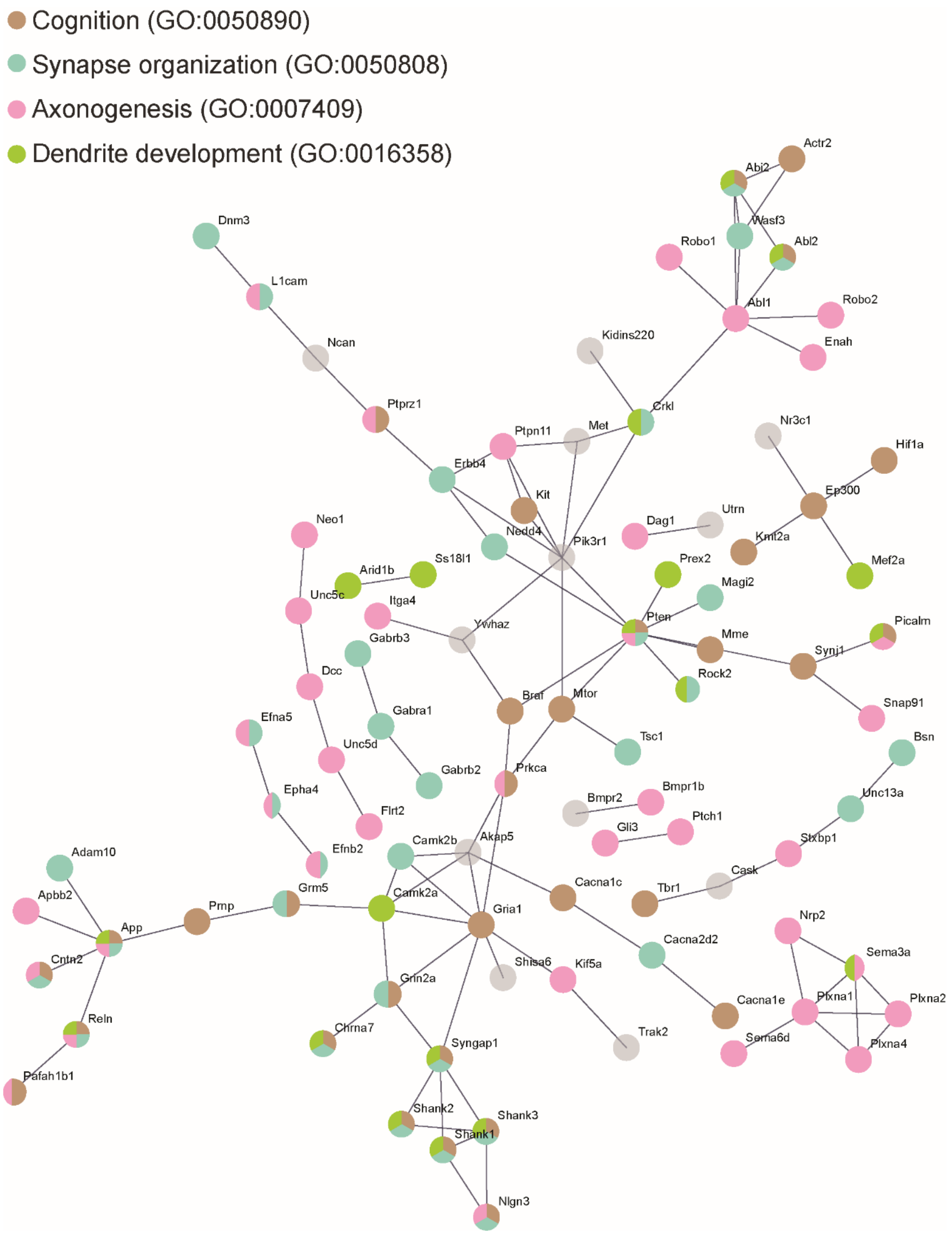

Supplementary Fig. 8: String network for the M3 module (inset in Fig. 3f). The legends in the upper part of the panel clarify their association with the respective pathways.

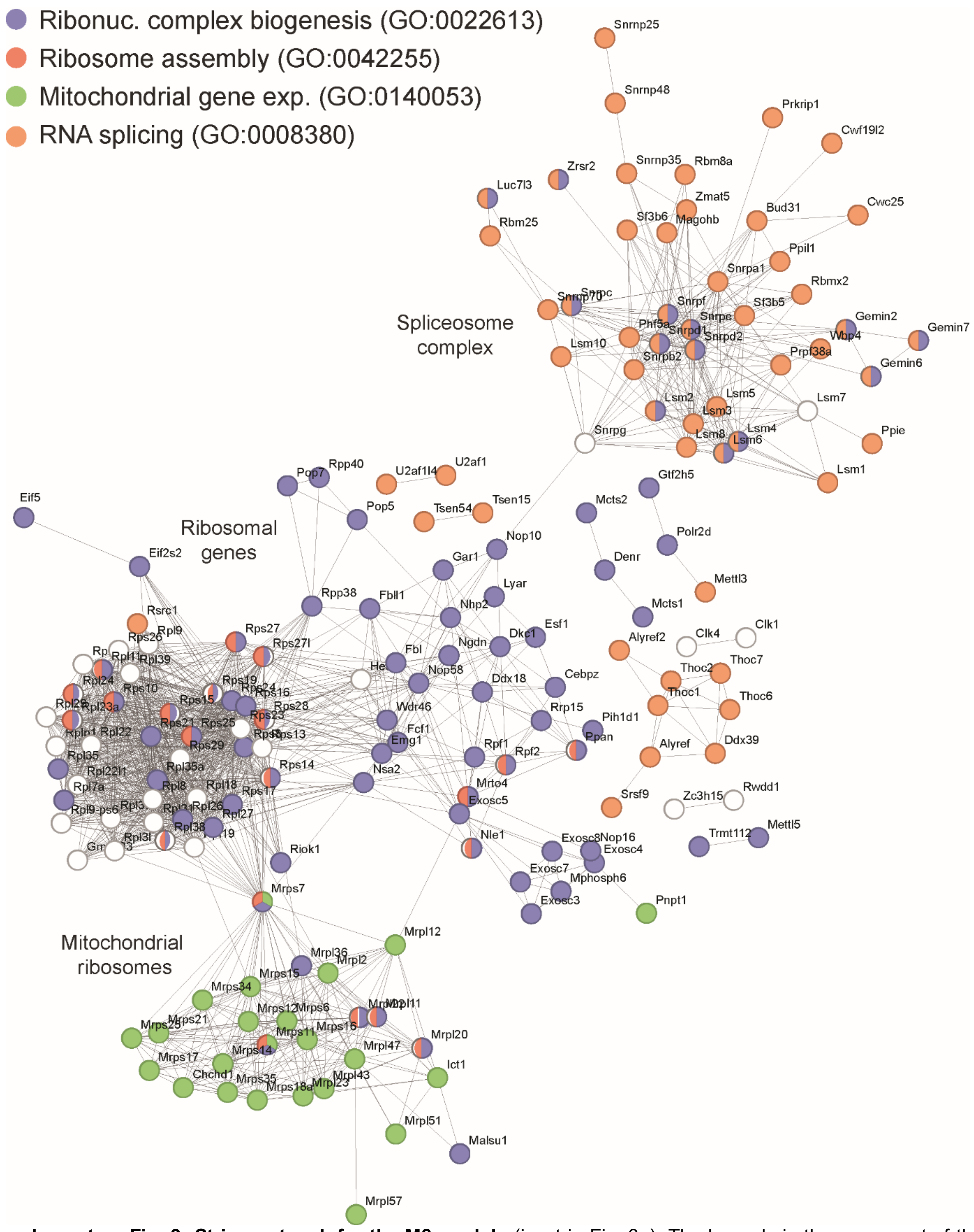

89 **Supplementary Fig. 9: String network for the M8 module** (inset in Fig. 3g). The legends in the upper part of the panel clarify their association with the respective pathways.

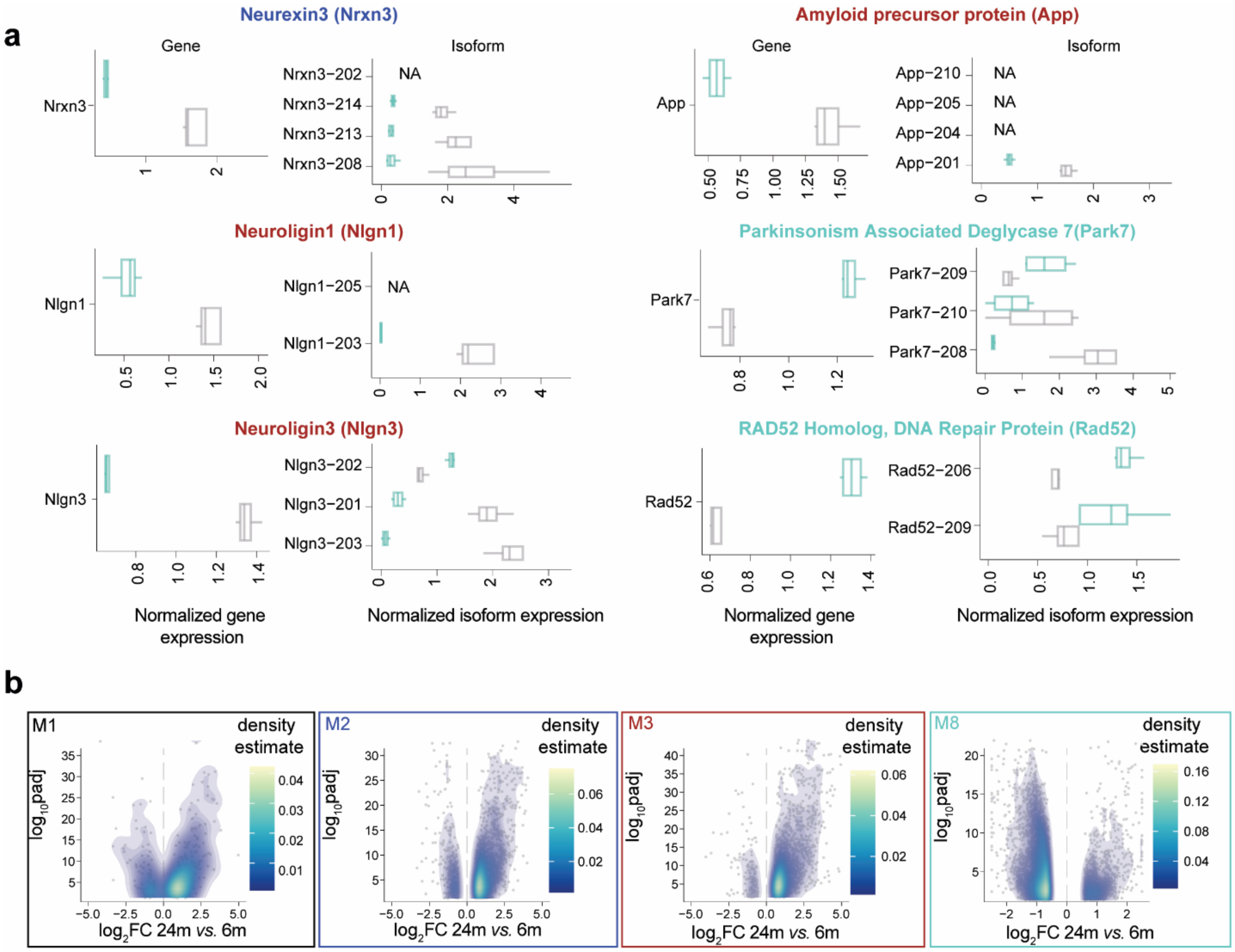

Supplementary Fig. 10: Cross-validation of the results in a new animal cohort. (**a**) Boxplot of the normalized expression for the sub-selection of genes shown in main **Fig. 4a** using replication of the results in a second independent animal cohort. Note also that the data from the second cohort of mice was processed and sequenced in a different sequencing facility. The panel on the left shows the gene expression while the right panels isoform expression. (**b**) Volcano density plots for the second unrelated mouse cohort of significantly differentially expressed mRNA isoforms within selected modules from the WGCNA analysis that show interesting patterns during brain aging (M1, M2, M3 and M8) similar to **Fig. 4b**.

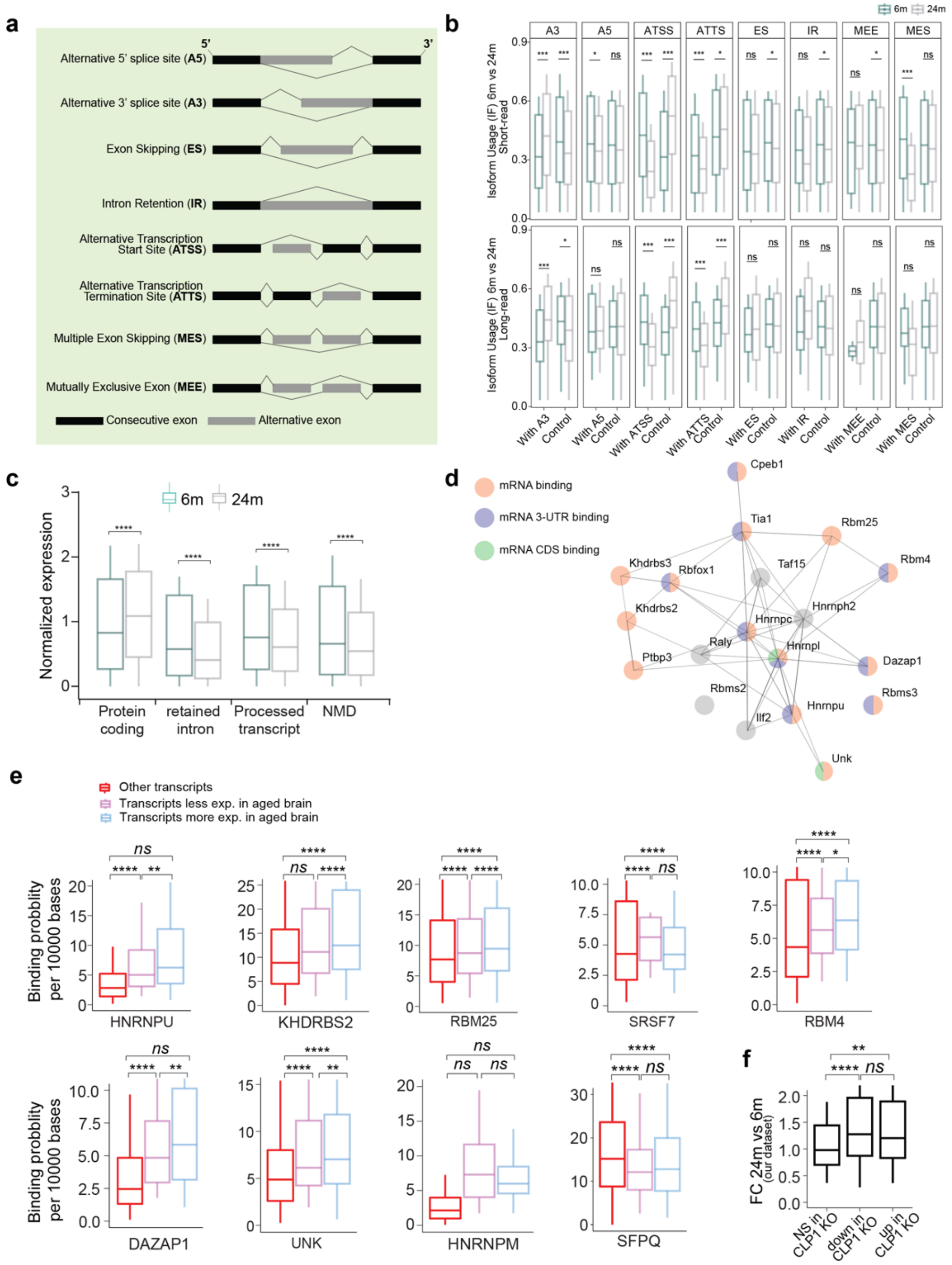

Supplementary Fig. 11: Alternative splicing events and binding probability analysis of RNA-binding proteins (RBPs) and splicing factors (SFs) for cDNA. (**a**) Types of alternative splicing events. **(b)** Isoform usage analysis for alternative splicing events in the short-read and long-read datasets. The significance test is performed using R's exact binomial test with default parameters and the resulting p-values are adjusted with adjusted p-value using FDR (Benjamini-Hochberg, * ≤ 0.05, ** ≤ 0.01, *** ≤ 0.001, and **** ≤ 0.0001). **(c)** Biotype designation of isoforms based on Ensembl (https://www.ensembl.org/) for those that are significantly differentially expressed in the Illumina dataset, genes were selected if they have at least 2 isoforms differentially expressed (*padj* ≤ 0.05 and $\log_2$FC ≥ |0.58|; paired t-test * ≤ 0.05, ** ≤ 0.01, *** ≤ 0.001, and **** ≤ 0.0001). **(d)** String network of RBPs curated from RBPmap (81). (**e**) Binding probability (calculated per 10'000bp) of RBPs and SFs on the cDNA and CLP1 (bottom right panel) for the genes that are significantly either down- or up-regulated in the CLP1 KO mice vs WT mice ((85) $\log_2$FC≥|0.58| and padj≤0.05; ANOVA followed by Tukey posthoc test * ≤ 0.05, ** ≤ 0.01, *** ≤ 0.001, and **** ≤ 0.0001). (**f**) Both gene groups that are either down- or up-regulated in CLP1 KO mice are significantly increased in our data when compared to genes not significantly changed in CLP1 KO (ANOVA followed by Tukey posthoc test ** ≤ 0.01, and **** ≤ 0.0001).

Log_2FC −0.8 0 0.8

**** ≤ 0.0001 | *** ≤ 0.001 | ** ≤ 0.01 | * ≤ 0.05 | – ≤ not significant

RBP	Down vs NS [Log_2FC]	Down vs NS [padj]	Up vs NS [Log_2FC]	Up vs NS [padj]
BOLL	0,05	*	0,00	–
CELF1	-0,14	–	-0,20	****
CNOT4	0,22	***	0,12	*
CPEB4	0,14	***	0,07	**
DAZ3	0,16	**	0,00	–
DAZAP1	0,13	–	0,20	–
EIF4G2	0,21	–	0,22	–
ELAVL4	-0,01	–	-0,15	*
ESRP1	0,47	****	0,33	***
EWSR1	0,28	****	0,11	*
FUBP1	-0,19	–	-0,26	****
FUBP3	-0,13	–	-0,23	****
FUS	0,46	****	0,45	****
HNRNPA0	0,00	–	-0,14	**
HNRNPA1	0,06	–	-0,08	–
HNRNPA2B1	0,20	****	0,14	–
HNRNPDL	-0,17	****	-0,31	–
HNRNPD	0,18	–	0,05	****
HNRNPF	0,27	****	0,19	****
HNRNPH2	0,24	**	0,37	**
HNRNPK	0,55	****	0,42	****
HNRNPL	0,05	**	0,05	–
HNRNPM	0,68	*	0,21	–
HNRNPU	0,10	–	0,17	–
IGF2BP1	0,01	**	-0,11	–
IGF2BP2	0,23	–	0,11	–
ILF2	0,41	****	0,27	–
KHDRBS2	0,26	****	-0,10	–
KHSRP	-0,11	–	-0,09	–
MBNL1	0,10	–	0,04	–
MSI1	0,10	***	0,07	–
NOVA1	0,00	–	0,01	–
NUPL2	0,16	****	0,02	–
PABPN1L	0,06	****	-0,12	–
PCBP1	0,54	****	0,39	****
PCBP2	0,31	****	0,22	****
PCBP4	0,27	–	0,13	–
PRR3	-0,08	–	-0,12	*
PTBP3	0,10	–	0,09	–
PUF60	0,09	****	0,08	–
PUM1	-0,16	****	-0,21	–
Ptbp1	-0,08	–	-0,02	****
RBM15B	-0,13	****	-0,19	*
RBM22	0,45	****	0,21	–
RBM23	0,59	–	0,56	***
RBM24	0,16	**	0,10	*
RBM25	0,36	****	0,32	****
RBM41	0,01	****	-0,09	–
RBM45	0,15	****	0,12	****
RBM47	-0,07	*	-0,16	–
RBM4B	0,28	–	0,16	–
RBM6	0,50	****	0,40	***
RBMS2	0,02	–	0,07	***
RBMS3	0,11	***	0,04	–
RC3H1	0,09	****	0,00	****
Rbfox1	0,37	–	0,23	–
Rbfox2	0,21	–	0,09	–
Rbm4	0,43	***	0,18	–
SF1	0,05	**	-0,05	–
SFPQ	0,05	–	-0,02	–
SNRPA	0,19	*	0,15	–
SRSF10	0,42	****	0,22	*
SRSF11	0,38	****	0,35	****
SRSF2	0,31	****	0,21	***
SRSF4	0,27	****	0,14	**
SRSF5	0,57	****	0,39	****
SRSF7	0,78	*	0,46	–
SRSF8	0,46	****	0,24	–
SRSF9	0,20	****	0,11	***
TAF15	0,44	*	0,26	–
TARDBP	0,13	****	0,03	–
TRA2A	0,26	–	0,21	–
TRNAU1AP	-0,08	–	-0,18	*
UNK	0,00	–	0,07	–
ZCRB1	0,05	*	0,02	–
ZFP36	0,05	****	-0,10	–
ZNF326	0,09	****	-0,02	–

Supplementary Fig. 12: Binding probability analysis of RNA-binding proteins (RBPs) and splicing factors (SFs) for 3'UTR. (**a**) Heatmap of the FC plotted as log_2 for the difference between the 24m and 6m by grouping transcripts that are either downregulated, upregulated, or non-significantly changed in the aged brain. Positive values (red) indicate a relatively higher binding probability in the selected group of genes and *padj* were calculated with ANOVA followed by Tukey post-hoc test (Supplementary Table 12; sheet name "RNA binding protein analysis for the 3'UTR2, p-value: * ≤ 0.05, ** ≤ 0.01, *** ≤ 0.001, and **** ≤ 0.0001)

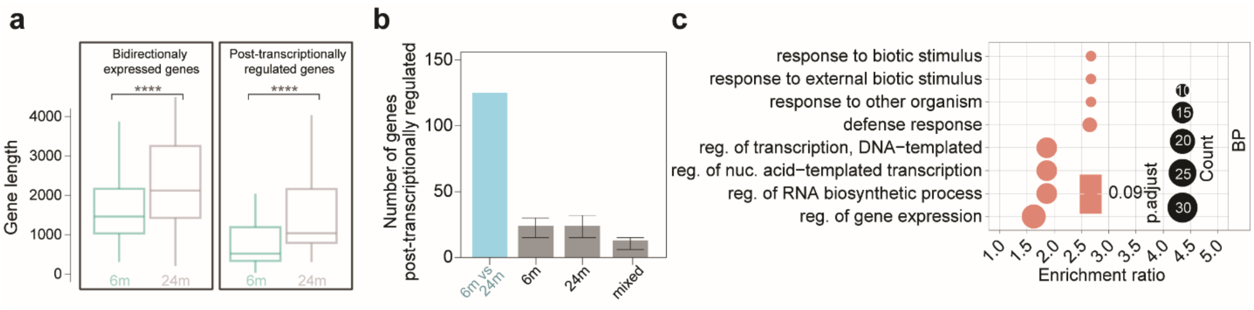

Supplementary Fig. 13: RNA dynamics comparison between 6m and 24m mice. **(a)** Boxplot of the length for genes that were bidirectionally expressed (left panel) and post-transcriptionally regulated (right panel; paired t-test, p-value: * ≤ 0.05, ** ≤ 0.01, *** ≤ 0.001, and **** ≤ 0.0001). (**b**) Number of genes identified as post-transcriptionally regulated comparing: the 24m samples against the 6m samples, each combination of two 6m samples against the remaining 6m samples (6m - null model 1), each combination of two 24m samples against the remaining 24m samples (24m - null model 2), each combination of two 6m samples and two 24m samples against the remaining ones (Mixed - null model 3). (**c**) Gene Ontology Biological Processes enriched in the set of genes post-transcriptionally regulated compared to the background (i.e., genes expressed in both the conditions); adjusted p-value threshold <= 0.05.
